# Different Blood Cell-Derived Transcriptome Signatures in Cows Exposed to Vaccination Pre- or Postpartum

**DOI:** 10.1371/journal.pone.0136927

**Published:** 2015-08-28

**Authors:** Rosemarie Weikard, Wiebke Demasius, Frieder Hadlich, Christa Kühn

**Affiliations:** Institute for Genome Biology, Leibniz Institute for Farm Animal Biology (FBN), Dummerstorf, Germany; The University of Melbourne, AUSTRALIA

## Abstract

Periparturient cows have been found to reveal immunosuppression, frequently associated with increased susceptibility to uterine and mammary infections. To improve understanding of the causes and molecular regulatory mechanisms accounting for this phenomenon around calving, we examined the effect of an antigen challenge on gene expression modulation on cows prior to (BC) or after calving (AC) using whole transcriptome sequencing (RNAseq). The transcriptome analysis of the cows’ blood identified a substantially higher number of loci affected in BC cows (2,235) in response to vaccination compared to AC cows (208) and revealed a divergent transcriptional profile specific for each group. In BC cows, a variety of loci involved in immune defense and cellular signaling processes were transcriptionally activated, whereas protein biosynthesis and posttranslational processes were tremendously impaired in response to vaccination. Furthermore, energy metabolism in the blood cells of BC cows was shifted from oxidative phosphorylation to the glycolytic system. In AC cows, the number and variety of regulated pathways involved in immunomodulation and maintenance of immnunocompetence are considerably lower after vaccination, and upregulation of arginine degradation was suggested as an immunosuppressive mechanism. Elevated transcript levels of erythrocyte-specific genes involved in gas exchange processes were a specific transcriptional signature in AC cows pointing to hematopoiesis activation. The divergent and substantially lower magnitude of transcriptional modulation in response to vaccination in AC cows provides evidence for a suppressed immune capacity of early lactating cows on the molecular level and demonstrates that an efficient immune response of cows is related to their physiological and metabolic status.

## Introduction

Particularly, the transition from pregnancy to lactation (comprising a period of 3–4 weeks before and after parturition) is marked by metabolic, hormonal and immunological changes that have an impact on the incidence of infectious and metabolic diseases of the cows [1]. It has been reported that periparturient cows undergo a period of immunosuppression of various immunological parameters associated with a high susceptibility to uterine and mammary infections (e.g., [2–6]).

Understanding the factors and mechanisms accounting for immunosuppression and disease incidence in periparturient cows is important to maintain the welfare, health and productivity of cows. Potential causal relationships between periparturient metabolism and immune function have been examined in numerous studies (e.g., [3,7–13]). Various hypotheses have been put forward that attribute the impairment of immune functions around the time of calving to endocrine or metabolic changes that appear to be linked to the initiation of milk production and related to the mobilization of endogenous body reserves to enable milk production. For instance, high non-esterified fatty acid (NEFA) concentrations observed in early lactation (e.g., [12]) are discussed to be associated with impaired lymphocyte proliferation and polymorphonuclear leukocyte functions [8,9,13]. Proliferation and IL4 secretion of peripheral blood mononuclear cells were found to be negatively correlated with the concentration of serum NEFA [8].

Although several recent studies have examined blood immunometabolic factors, hematological and peripheral blood leukocyte and neutrophil expression profiles of cows during the periparturient period (e.g., [8,14–16]), the causes and mechanisms of the immunosuppression phenomenon around calving are not completely understood. The effect of antigen challenge on the modulation of gene expression in cows vaccinated prior to or after calving has not been examined in detail.

RNA-based next-generation sequencing (RNAseq) provides a tremendous amount of new information regarding gene expression, transcript structure, function and regulation on a whole transcriptome level. Comparative transcriptional profiling of bovine blood leucocytes and macrophages in response to infection using RNAseq and gene expression microarrays showed that RNAseq is superior in detecting differential gene expression because of its increased dynamic range [17,18]. Recently, we demonstrated that RNAseq analysis is remarkably applicable for monitoring the immune response in whole blood of cows subjected to an antigen challenge [19]. The results of our study provided a comprehensive catalogue of transcripts expressed in whole bovine blood and identified transcripts differentially expressed in response to the antigen challenge.

The focus of this study was to elucidate the influence of the antigen challenge on the transcriptional modulation of the immune response in cows differing regarding lactation status to monitoring the regulatory transcriptional processes reflected in the whole blood of these cows. Therefore, we performed a follow-up investigation based on our previous whole transcriptome RNAseq dataset [19]. We hypothesized that an efficient immune response to antigen challenge is dependent on the specific metabolic status of the cows around calving and postulated that differences in the immune response are reflected at transcriptional level in blood cells of pre- and post-calving cows.

## Materials and Methods

### Animals and sampling

Animals, sampling and data collection were identical to those described in our previous study [19]. The study included six lactating and six non-lactating cows (Table 1). Except for one Holstein cow, all individuals were F_2_ cows from a German Holstein x Charolais crossbred population [20]. All cows were before or after second/third parturition except for one pregnant heifer from the German Holstein x Charolais population. The cows were kept under identical dairy cow conditions on the experimental farm of the FBN Dummerstorf. All cows had received a basic double vaccination with an inactivated vaccine (PregSure, Pfizer) against the Bovine Virus Diarrhoea Virus according to the manufacturer’s recommendations and at least one booster vaccination 15 months prior to our experiment. Jugular blood was taken immediately prior a further booster vaccination with PregSure and 14 days after vaccination. After sampling, 2.5 ml blood was immediately transferred to PAXgene blood RNA tubes (PreAnalytiX, Hombrechtikon, Switzerland). Blood samples were frozen and stored at -80°C until further processing.

**Table 1:** Characteristics of cow groups formed by cluster analysis.

Cow_ID	Origin	Number of calvings	Lactationstatus	Vaccination day relative to calving	Group
1	CH x GH	1	dry	-120	BC
7	CH x GH	1	dry	-48	BC
8	CH x GH	1	dry	-120	BC
11	CH x GH	1	dry	-111	BC
2	CH x GH	2	early	+11	AC
3	CH x GH	2	early	+18	AC
6	CH x GH	2	early	+10	AC
10	CH x GH	2	early	+14	AC
12	CH x GH	2	early	+13	AC
4	CH x GH	2	mid	+82	ex
9	CH x GH	0	dry	-97	ex
5	GH	3	dry	-201	ex

Charolais x German Holstein F_2_ (CH x GH), purebred German Holstein (GH), AC: after calving, BC: prior to calving, ex: no group assignment, cows were excluded from subsequent data analysis due to the results of the cluster analysis

### Ethics statement

All experimental procedures were carried out according to the German animal care guidelines and were approved and supervised by the relevant authorities of the State Mecklenburg-Vorpommern, Germany (State Office for Agriculture, Food Safety and Fishery Mecklenburg-Western Pommerania (LALLF M-V), 7221.3–2.1-005/11).

### Library preparation, sequencing, sequence assembly and locus annotation

Preparation and sequencing of the libraries as well as all bioinformatic analyses comprising sequence assembly and locus annotation were performed as described previously [19]. Briefly, whole blood total RNA was extracted using the PAXgene Blood RNA Kit (PreAnalytiX, Hombrechtikon, Switzerland) according to the manufacturer’s instructions. Elimination of residual genomic DNA and RNA quality evaluation were carried out as described previously [19]. For each individual, two RNAseq libraries were prepared from samples collected before and 14 days after booster vaccination. Libraries indexed by different adaptors were prepared from 1 μg total RNA applying the Illumina TruSeq RNA library preparation kit (Illumina, San Diego, USA). After quality control, indexed libraries were pooled, and paired-end sequencing (61 cycles for each end) was performed on a Genome Analyser GAIIx (Illumina, San Diego, USA).

After demultiplexing of reads and FastQC quality check, reads were aligned to the bovine reference genome UMD3.1 [21] using Bowtie/TopHat 2.03 options [22] and guided alignment options supplying TopHat (TopHat—Genome sequence indices, http://tophat.cbcb.umd.edu/igenomes.shtml) with the bovine gene model annotation from Igenome (http://cufflinks.cbcb.umd.edu./igenomes.html, NCBI version). The resulting BAM file from read alignments was subjected to mismatch and multiple read filtering steps using SAMtools [23], and the filtered BAM file was submitted to transcript assembly using Cufflinks 2.02 options [22] and merged with the Igenome reference annotation and the bovine Ensembl gene annotation release 66 (Ensembl genome repository, Bos taurus release 66, ftp://ftp.ensembl.org/pub/release-66/gtf/). The resulting final gtf-file served for locus and transcript quantification using Cuffdiff 2.02 with the bovine UMD3.1 as reference genome assembly.

### Cluster analysis and differential expression analysis

To examine the transcriptional response to vaccination, a cluster analysis including all 12 individuals (Table 1) was performed. The read counts per locus and sample were extracted from the final annotation file applying the Cuffdiff options. This dataset served as input for the cluster analysis and the subsequent differential expression analysis. The parameter for the cluster analysis was the normalized difference (in counts per million reads, cpm) between pre and post-vaccination sampling for each individual and each locus. Only loci with a minimum expression > 1 cpm in each of the 24 samples were included. Cluster analysis was performed using PermutMatrix [24] for calculation of Euklidian Distance applying McQuitty’s criteria for linkage rule and Multiple-fragment heuristic (MF) for Tree Seriation rule.

Differential expression analysis was performed using the EdgeR algorithm [25]. After calculation of the normalization factor, the effect on gene expression was calculated applying the model: counts = individual + treatment. Multiple testing by calculating the false discovery rate (FDR) according to [26] was accounted for when testing for statistical significance. Differences in expression with a significance threshold of q< 0.05 were considered statistically significant.

### Pathway analysis

Ingenuity pathway analysis (IPA, Ingenuity Systems, http://www.ingenuity.com) was applied to identify biological functions, canonical pathways and networks involved in the response to vaccination in pre- and post-calving cows. Only annotated loci with a statistically significant differential vaccination-induced expression level (q< 0.05) were included in the pathway analyses. For IPA analysis, the respective log _fold change_ (logFC) of differentially expressed genes prior to and after vaccination was used to indicate the direction and quantity of differential expression.

Gene Ontology (GO) analysis on RNAseq data (GOseq analysis, [27]) was used to identify over- or underrepresentation of differentially expressed genes in the set of GO terms and pathways of the KEGG (Kyoto Encyclopedia of Genes and Genomes) database (http://www.genome.jp/kegg/pathway.html). The analysis was performed by referring to the human Ensembl annotation because of the incomplete functional annotation of the bovine genome. The length bias characteristic for RNAseq data was accounted for using the Wallenius distribution to approximate the true null distribution. The respective p-value for over- or underrepresented pathways was calculated from the null distribution.

## Results and Discussion

Demultiplexing and filtering yielded 57.8–79.7 million reads per sample, 85.1–89.0% of which were mapped on the bovine genome. Detailed results regarding read mapping, sequence assembly, locus annotation and transcript quantification were presented in detail in our previous report [19]. The results presented here focus on the different response of the cows on transcriptome level in whole blood with a specific emphasis placed on the different time point of antigen challenge relative to calving, pre- or postpartum.

### Cluster analysis

**Fig 1:**
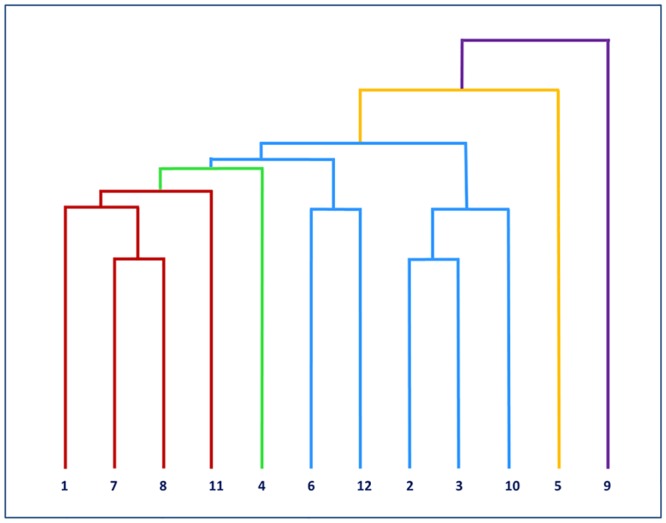
Cluster analysis of transcriptional response to vaccination across all cows included in the RNAseq experiment. Red: non-lactating cows vaccinated prior to calving (BC), blue: early lactating cows vaccinated after calving (AC), green: mid- lactating cow, yellow: non-lactating cow of different genetic background, lavender: non-lactating first calving cow.

The hypothesis-free cluster analysis revealed a clear separation of cow 9 from all other cows (Fig 1), which could be due to the fact that cow 9 is the only individual investigated prior to first calving. All other cows had experienced at least a single parturition (Table 1). Furthermore, cow 5, a purebred German Holstein cow, segregated separately (Fig 1), which is obviously owing to its different breed origin (GH), whereas the genetic background of all other individuals in the experiment is a Charolais x German Holstein (CH x GH) F_2_ cross (Table 1). In addition to these two divergent individuals, another group of four cows from the CH x GH cross (cows 1, 7, 8, 11) can be distinguished within the remaining main group of 10 cows (Fig 1). Common feature of those four cows is that at vaccination, they did not lactate and were 7 to 18 weeks prior to their second calving (Table 1). The remaining cows had delivered their second calf prior to vaccination. Five cows (cows 2, 3, 6, 10, 12) were in early lactation and had given birth to a calf about two weeks before vaccination. Cow 4 was in mid lactation and had delivered a calf 12 weeks before vaccination.

Thus, the hypothesis-free cluster analysis identified those cows that had a different genetic background or had not experienced calving before resulting in clustering of cows according to their stage of lactation and time point of vaccination relative to calving. For subsequent analysis of vaccination effect on the transcriptome with respect to the calving event, we restricted our dataset to nine cows as indicated in Table 1. We compared individuals clustered in the two groups of cows from the CH x GH cross population, which differed in vaccination timing: before calving (BC, non-lactating) and after calving (AC, early lactating). At the time point of vaccination, the four BC cows were at 6.9–17.1 weeks prior to calving, and the five AC cows had delivered a calf 1.4–2.6 weeks before calving. These two cow groups were subsequently investigated regarding their whole transcriptome response to vaccination. The respective data was extracted from the dataset and re-analyzed according to the respective group design (Table 1).

### Different magnitude of transcriptional response to vaccination in pre- and post-calving cows

**Fig 2:**
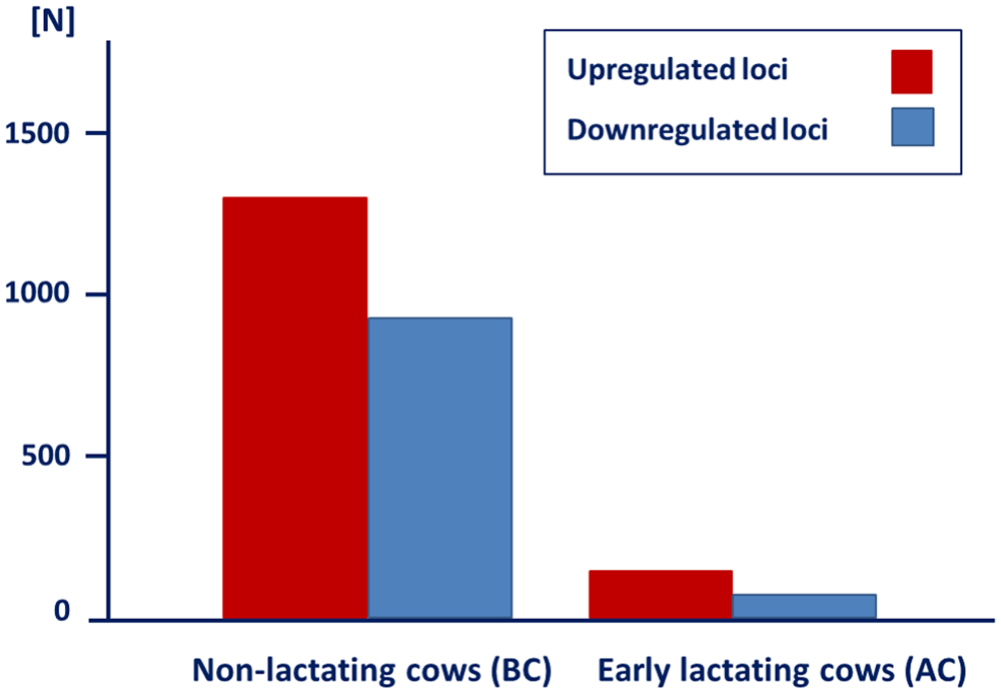
Number of differentially expressed loci in cows vaccinated prior to and after calving. N: Number of differentially expressed transcripts, BC: Cows vaccinated prior to calving, non-lactating. AC: Cows vaccinated prior to calving, early lactating.

The analysis of the whole transcriptome modulation in blood in response to vaccination indicated 208 differentially expressed loci in the AC cows vaccinated after calving (q<0.05, Table 2, Fig 2, S1 Table). Out of them, 68 loci were downregulated and 140 loci were upregulated in response to vaccination. Notably, nearly half of the 208 loci differentially expressed had no annotation in the bovine reference genome assembly (Table 2). Compared to the AC cows, the number of differentially expressed loci in response to vaccination was about 10-fold higher in the BC cows vaccinated prior to calving. In this group, the transcription levels of a total of 2,235 loci were significantly modulated after vaccination (q<0.05, Table 2, Fig 2, S2 Table); out of which 936 loci were downregulated whereas 1,300 loci were upregulated. In the BC group, a markedly lower percentage of significantly affected loci (7%, 149 loci) were unknown, whereas 2,087 loci were annotated in the bovine reference genome database (Table 2).

**Table 2:** Summary of number of loci modulated on expression level in response to vaccination in cows prior to and after calving.

Number (q< 0.05)	AC	BC	AC+BC intersection
**Affected loci in total**	208	2,235	47
**Annotated**	106	2,087	37
**Unannotated**	102	149	10
**Upregulated**	140	1,300	44/43
**Downregulated**	68	936	3/4

AC: vaccination after calving, BC: vaccination prior to calving

The intersection of loci modulated in response to vaccination in both cow groups comprised a total of 47 loci (q<0.05, Table 2). This common set of genes (S3 Table), the majority of which was upregulated in response to vaccination in both cow groups, might represent loci that are of general functional relevance due to antigen response, independent of vaccination time point, pre- or postpartum, and independent of lactation status, non- or early lactating.

The higher magnitude of transcriptional response to vaccination in the BC group compared to cows of the AC group provides evidence for a generally reduced reactivity of the cows vaccinated at about two weeks postpartum and suggests that the specific response to vaccination elicits a divergent regulatory immune defense reaction in each group. The early lactating AC cows are in a physiological stage that is associated with metabolic changes due to the onset of lactation. In dairy cows, this stage is frequently associated with a shifting of the metabolism into a severe negative energy balance, as the energy demands for lactogenesis exceed energy intake (e.g., [28–30]). Coping with these considerable metabolic challenges associated with lactation is of first priority for cows in the early lactation period. It has been documented in several studies that this intense metabolic burden may have a negative impact on immune functions of cows in the periparturient period [4,7,9–11,31,32]. Wathes et al. [15] showed that global gene expression and immune response were altered in cows with severe negative energy balance and concluded that uterine involution and elimination of contaminant bacteria will be delayed in cows with negative energy balance post calving.

In contrast to high-yielding dairy cows, the CH x GH cows investigated in our vaccination experiment are characterized by a relatively low milk yield [33–35]. Thus, a high fat mobilization and negative energy balance postpartum should be less relevant in this meat x dairy cross population compared to high yielding Holstein dairy cows, which display a higher genetic merit for milk production and show often a strong negative energy balance in early lactation [36–38]. Consequently, other physiological mechanisms than a disturbed energy balance should be affected in the early lactating CH x GH cows of our study.

In order to determine the biological relevance of the modulation of the identified differentially expressed genes in both cow CH x GH groups in response to vaccination, we performed functional enrichment analyses applying different tools and pathway databases as the Ingenuity Pathways Analysis (IPA) software, the Gene Ontology (GO) terms and KEGG (Kyoto Encyclopedia of Genes and Genomes) pathways. The results of these analyses enable the functional classification of differentially expressed genes, biological functions and pathways divergently regulated in response to vaccination and between the cow groups.

### Differentially enriched biological processes in response to vaccination in pre- and post-calving cows

The functional enrichment analysis based on GO categories showed that in the BC cows the absolute number of significantly (p<0.05) affected GO terms was substantially higher compared to the AC cows. In total, 294 GO categories were significantly overrepresented in response to vaccination in BC cows (S4 Table). The most significantly enriched GO terms in this cow group comprise a variety of biological processes included in the regulation of gene expression and protein biosynthesis, particularly mRNA translation, ribosome structure and expression. Furthermore, biological processes associated with mechanisms focusing on catabolic processes (nonsense-mediated mRNA decay, RNA catabolic process, macromolecular complex disassembly) and on biological processes involved in virus survival (viral life cycle, viral genome expression and transcription, viral infectious cycle) were found to be enriched in response to vaccination. A downregulation of translation in response to a viral attack was reported to operate against a number of viruses [39,40] in order to protect the individual against virus replication [41]. The host cell protein synthesis shut-off has been discussed as an antiviral protection pathway in our previous study [19]. In addition, several GO terms enriched in the electron transport chain pathway were found significantly enriched in the BC cows (e.g., NADH dehydrogenase complex, NADH dehydrogenase (ubiquinone) activity, mitochondrial ATP synthesis coupled electron transport, oxidative phosphorylation) as well as biological processes focusing on a variety of immune defense mechanisms such as antigen processing and presentation, positive regulation of chronic inflammatory response to antigenic stimulus and tumor necrosis factor receptor binding; see, S4 Table).

In the AC group, we found 36 GO categories to be significantly overrepresented in response to vaccination (S5 Table). The most significantly affected GO terms in the AC cow group showed a focus on the development and differentiation of blood cells including biological processes associated with myeloid cell and erythrocyte differentiation, hematopoiesis, hemoglobin complex as well as gas transport and oxygen gas transport. In addition, biological processes related to cellular stress (cellular response to hydrogen peroxide) and to the immune response like immune system process and development and cytokine receptor activity and complement component C1 complex (S5 Table) were enriched in the AC cows in response to vaccination. In contrast to the BC cow group, we did not see an enrichment of processes involved in the regulation of protein biosynthesis in the AC cows, indicating that in the early lactation period, cows are obviously incapable to activate the antiviral pathway of protein synthesis downregulation for protection against viral attack.

### Biological functions affected by vaccination prior and after calving

**Fig 3:**
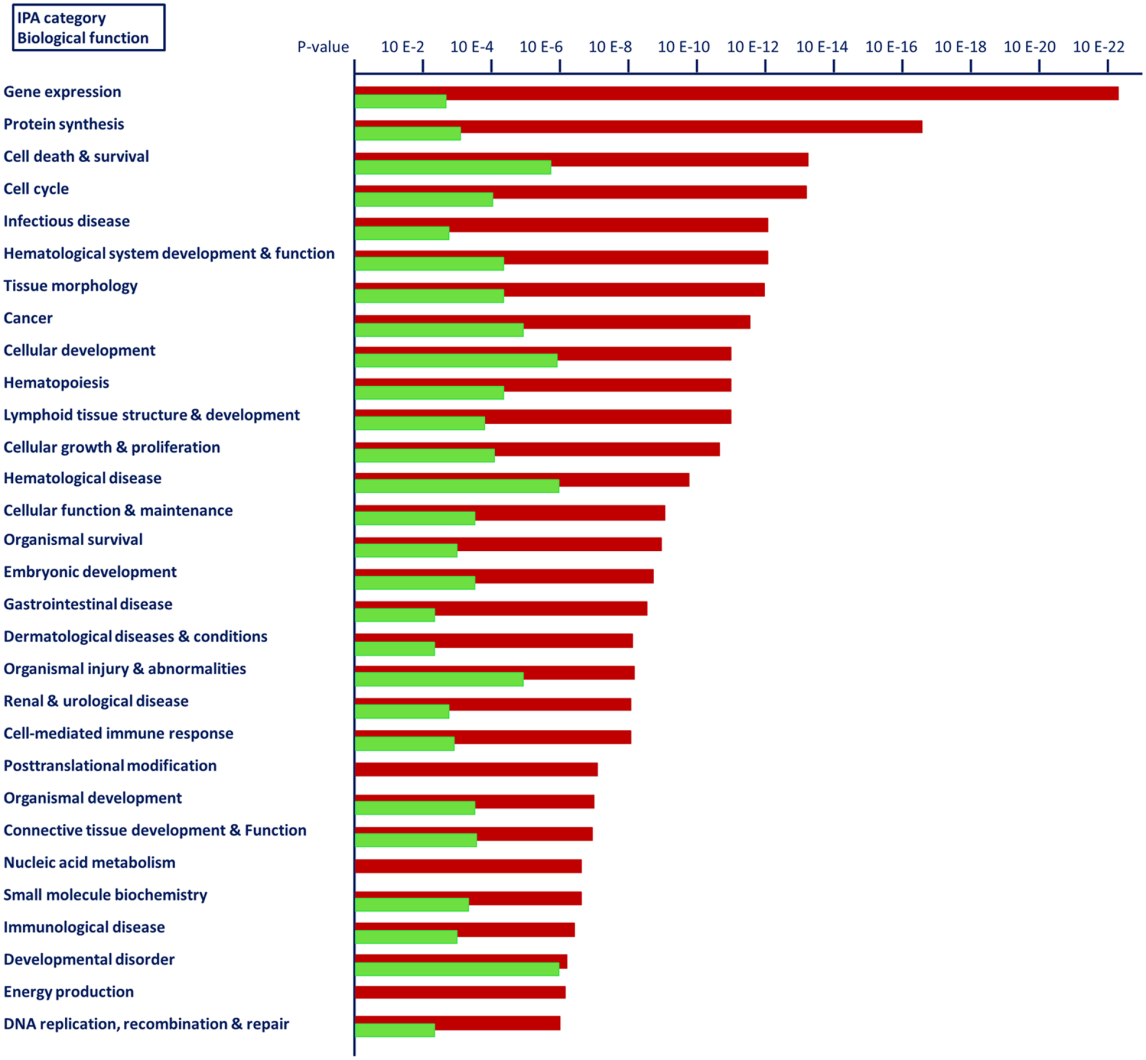
The most significant biological function categories (IPA) affected in cows vaccinated prior to and after calving. Vaccination: Red, prior to calving (BC); green: after calving (AC).

Analysis of biological functions overrepresented by differentially expressed genes due to vaccination using the IPA software clearly demonstrates that there is a highly divergent response of both cow groups on the transcriptional level in response to antigen challenge (Fig 3). This profile reflects the results obtained for modulated biological processes in the GO term enrichment analysis. In response to antigen challenge both, the number of affected biological functions and the level of significance were substantially higher in the BC group compared to the AC group. The IPA analysis revealed that there are some similar main functional categories affected in both cow groups, like differentiation and development of the blood cell system, cellular development, death and survival, immune cell response, signaling and trafficking. In the BC cows, a total of 59 biological function categories showed to be overrepresented, whereas in the AC cow group, 34 categories displayed enrichment (p<0.001, S6 and S7 Tables). Noticeably, biological functions associated with gene and protein expression, cell death and survival and infectious disease were most significantly affected in BC cows. For the AC cows, biological functions related to cellular development, hematological disease and cell death and survival were the most significantly affected (Fig 3, S7 Table).

In concordance with results from the functional enrichment analysis of GO terms, the results of the IPA analysis indicate that the intrinsic functional ability to respond to antigen challenge seems to be suppressed in cows that are in the critical period post calving. The generally reduced transcriptional response in the AC cows is most probably ascribed to the fact that the priority of cows in the early lactation interval is to cope with physiological challenges due to physical recovery and reorganization events required postpartum and to metabolic stress due to the onset of lactation. Additional burden due to the intervention in the immune system by exposure to vaccination cannot be defended adequately in the AC cows compared to the BC cows. Thus, the results of our whole transcriptome analysis suggest that the immune defense system of the AC cows might be suppressed after calving as has been reported in literature for cows in the early lactation period.

### KEGG pathways modulated by differently expressed genes in response to vaccination

The KEGG pathway analysis revealed that a spectrum comprising 35 biological KEGG pathways was affected in BC cows (S8 Table). In concordance with the GO enrichment analysis, we found a superior overrepresentation of gene sets enriched in the ribosome pathway indicating again the modulation of protein synthesis in response to vaccination. In addition, RNA and protein degradation RNA transport and protein export are categories that were consistently enriched in both enrichment analyses. Moreover, highly significant vaccination-induced enrichment of a variety of KEGG disease categories was identified in the BC cow group (e.g., neuronal diseases, carcinoma, myeloid leukemia) as well as pathways focusing on processes involved in programming of immune response (e.g., T cell and B receptor signaling, natural killer cell mediated cytotoxicity, bacterial invasion of epithelial cells, chemokine signaling). Interestingly, KEGG pathway analysis highlighted pathways important in energy metabolism, like oxidative phosphorylation and carbohydrate digestion and absorption. In contrast to the BC cows, vaccination-induced gene set enrichment analysis in the AC cows revealed only 11 significantly overrepresented KEGG pathways. (S9 Table). On the top of the list of loci enriched in the AC group are categories comprising processes relevant for immune signaling (complement and coagulation cascades, hematopoietic cell lineage and JAK-STAT system) but also processes involved in oxidative phosphorylation and the excretory system associated with gas exchange (proximal tubule bicarbonate reclamation, collecting duct acid secretion).

### Canonical pathways enriched by differentially expressed genes in response to vaccination

Using IPA analysis, a total of 195 canonical pathways were significantly enriched by the genes that were differentially regulated in the BC cow group (p<0.05, S10 Table) in response to vaccination. Out of them, a total of 36 pathways were found to be most significantly affected in this group (p<5.0E-05, Table 3). In contrast to the BC cow group, the number and the level of significance of canonical pathways affected in the AC cow group are substantially lower. A total of 17 canonical pathways were significantly (p<0.05) enriched by the genes that were differentially regulated in AC cows (Table 4).

**Table 3:** The canonical pathways most significantly enriched by differentially expressed genes in response to vaccination prior to calving.

Canonical Pathway	P-value	Ratio	Down-regulated	Up-regulated
EIF2 Signaling	7.94E-45	5.31E-01	76/192 (40%)	26/192 (14%)
Regulation of eIF4 and p70S6K Signaling	7.94E-22	3.99E-01	41/163 (25%)	24/163 (15%)
mTOR Signaling	2.51E-19	3.65E-01	42/197 (21%)	30/197 (15%)
Mitochondrial Dysfunction	3.98E-15	3.84E-01	47/138 (34%)	6/138 (4%)
FLT3 Signaling in Hematopoietic Progenitor Cells	6.03E-09	3.82E-01	5/76 (7%)	24/76 (32%)
Glucocorticoid Receptor Signaling	2.82E-08	2.32E-01	19/280 (7%)	46/280 (16%)
Prolactin Signaling	8.32E-08	3.51E-01	3/77 (4%)	24/77 (31%)
JAK/Stat Signaling	8.51E-07	3.43E-01	3/70 (4%)	21/70 (30%)
Mouse Embryonic Stem Cell Pluripotency	8.91E-07	3.03E-01	5/99 (5%)	25/99 (25%)
Huntington’s Disease Signaling	1.58E-06	2.26E-01	17/234 (7%)	36/234 (15%)
Protein Ubiquitination Pathway	1.74E-06	2.23E-01	28/264 (11%)	31/264 (12%)
PI3K/AKT Signaling	1.78E-06	2.55E-01	8/137 (6%)	27/137 (20%)
Renal Cell Carcinoma Signaling	2.09E-06	3.33E-01	4/72 (6%)	20/72 (28%)
Erythropoietin Signaling	3.31E-06	3.11E-01	4/74 (5%)	19/74 (26%)
Estrogen Receptor Signaling	3.98E-06	2.61E-01	12/134 (9%)	23/134 (17%)
NF-kB Activation by Viruses	4.90E-06	3.04E-01	4/79 (5%)	20/79 (25%)
Melanoma Signaling	5.01E-06	3.86E-01	3/44 (7%)	14/44 (32%)
Thrombopoietin Signaling	5.25E-06	3.39E-01	2/59 (3%)	18/59 (31%)
Growth Hormone Signaling	5.89E-06	3.19E-01	1/72 (1%)	22/72 (31%)
CNTF Signaling	8.32E-06	3.45E-01	2/55 (4%)	17/55 (31%)
Endometrial Cancer Signaling	8.32E-06	3.45E-01	3/55 (5%)	16/55 (29%)
IL3 Signaling	1.00E-05	3.15E-01	3/73 (4%)	20/73 (27%)
VEGF Signaling	1.12E-05	2.78E-01	6/97 (6%)	21/97 (22%)
IGF1 Signaling	1.38E-05	2.75E-01	5/102 (5%)	23/102 (23%)
Prostate Cancer Signaling	1.41E-05	2.69E-01	5/93 (5%)	20/93 (22%)
ERK5 Signaling	1.48E-05	3.28E-01	3/64 (5%)	18/64 (28%)
GM-CSF Signaling	1.48E-05	3.13E-01	3/67 (4%)	18/67 (27%)
Integrin Signaling	1.55E-05	2.22E-01	8/207 (4%)	38/207 (18%)
Molecular Mechanisms of Cancer	2.40E-05	1.88E-01	12/367 (3%)	57/367 (16%)
Hereditary Breast Cancer Signaling	2.69E-05	2.50E-01	9/124 (7%)	22/124 (18%)
FAK Signaling	3.47E-05	2.55E-01	2/98 (2%)	23/98 (23%)
Acute Myeloid Leukemia Signaling	4.37E-05	2.84E-01	5/81 (6%)	18/81 (22%)
IL2 Signaling	4.47E-05	3.21E-01	3/56 (5%)	15/56 (27%)
Rac Signaling	4.68E-05	2.39E-01	5/117 (4%)	23/117 (20%)
B Cell Receptor Signaling	4.68E-05	2.32E-01	6/164 (4%)	32/164 (20%)
Myc Mediated Apoptosis Signaling	4.90E-05	3.17E-01	6/60 (10%)	13/60 (22%)

Functional enrichment pathway analysis for the set of genes differentially regulated by vaccination was determined using the IPA software. The ratio provides the proportion of genes in the respective canonical pathway, which are significantly differentially expressed after vaccination, relative to all genes present in the pathway. The proportion of upregulated and downregulated genes in the respective pathway is indicated. The table provides the pathways most significantly modulated (statistical significance: p<5.0E-05). A full list of differentially expressed genes assigned to the pathway is provided in S10 Table including all pathways modulated in response to vaccination with a statistical significance threshold of p<0.05.

**Table 4:** The canonical pathways most significantly enriched by differentially expressed genes in response to vaccination after calving.

Canonical Pathway	P-value	Ratio	Down-regulated	Up-regulated	Molecules
Complement System	1.26E-04	1.18E-01	0/34 (0%)	4/34 (12%)	CD59, C1QC, C1QA, C1QB
RhoA Signaling	2.29E-03	4.27E-02	1/117 (1%)	4/117 (3%)	LPAR6, ACTA2, PPP1CB, PIP5K1B, MYL3
Glycogen Degradation II	2.63E-03	2.00E-01	0/10 (0%)	2/10 (20%)	PYGM, PGM5
Glycogen Degradation III	3.80E-03	1.67E-01	0/12 (0%)	2/12 (17%)	PYGM, PGM5
Regulation of Actin-based Motility by Rho	3.98E-03	4.65E-02	1/86 (1%)	3/86 (3%)	ACTA2, PPP1CB, PIP5K1B, MYL3
Pattern Recognition Receptors in Recognition of Bacteria and Viruses	6.92E-03	4.00E-02	0/100 (0%)	4/100 (4%)	OAS1, C1QC, C1QA, C1QB
Primary Immuno-deficiency Signaling	7.94E-03	4.84E-02	1/62 (2%)	2/62 (3%)	IL7R, ZAP70, IGHD
L-serine Degradation	2.34E-02	3.33E-01	1/3 (33%)	0/3 (0%)	SDSL
Hepatic Fibrosis / Hepatic Stellate Cell Activation	2.40E-02	2.82E-02	1/142 (1%)	3/142 (2%)	IGFBP4, IL1RL1, ACTA2, MYL3
B Cell Development	2.63E-02	6.06E-02	1/33 (3%)	1/33 (3%)	IL7R, IGHD
IL-9 Signaling	2.95E-02	5.26E-02	0/38 (0%)	2/38 (5%)	IL9R, SOCS2
Role of JAK2 in Hormone-like Cytokine Signaling	2.95E-02	5.56E-02	0/36 (0%)	2/36 (6%)	GHR, SOCS2
Arginine Degradation I (Arginase Pathway)	3.09E-02	2.50E-01	0/4 (0%)	1/4 (25%)	ARG1
Tetrapyrrole Biosynthesis II	3.89E-02	2.00E-01	0/5 (0%)	1/5 (20%)	ALAS2
Urea Cycle	4.68E-02	1.67E-01	0/6 (0%)	1/6 (17%)	ARG1
Arginine Degradation VI (Arginase 2 Pathway)	4.68E-02	1.67E-01	0/6 (0%)	1/6 (17%)	ARG1
GDP-glucose Biosynthesis	4.68E-02	1.67E-01	0/6 (0%)	1/6 (17%)	PGM5

Functional enrichment pathway analysis for the set of genes differentially regulated by vaccination was determined using IPA software. Statistical significance: (p<0.05). The ratio provides the proportion of genes in the respective canonical pathway, which are significantly differentially expressed after vaccination, relative to all genes present in the pathway. The proportion of upregulated and downregulated genes in the respective pathway and a full list of differentially expressed genes (molecules) assigned to the pathway are provided.

#### Protein biosynthesis and posttranslational processes are downregulated in cows vaccinated prior to calving

Ingenuity canonical pathway analysis in the BC group highlighted the EIF2 signaling and EIF4 and p70S6K signaling as most significantly enriched pathways (Table 3, S10 Table) in response to vaccination indicating a strongly modulated protein biosynthesis on the step of translational regulation, which is also supported by the functional enrichment analyses of GO terms and KEGG pathways (S4 and S8 Tables). IPA analysis showed that these two pathways are represented by numerous affected genes, of which 14% were upregulated and 40% were downregulated. Out of the 30 most significantly vaccination-affected loci in the BC group (S2 Table), eight loci encode ribosomal proteins (*RPL34*, *RPL37A*, *RPL27*, *RPL31*, *RPL38*, *RPS21*, *RP36AL*, *RPL21*) and additional five loci are related to them (*LOC100848726*, *LOC784243*, *LOC782021*, *LOC100296205*, *LOC790088*). Concordantly, all of them were highly significantly downregulated after vaccination in BC cows (S2 Table). The substantial downregulation of protein biosynthesis in the BC group is in agreement with the upregulation of *DR1* (S2 Table), the down-regulator of transcription and negative cofactor of TATA box protein binding, which is known to inhibit the assembly of the RNA polymerase II preinitiation complex [42]. A vaccination-induced downregulation of the protein biosynthesis process was not observed in AC cows. Details regarding downregulation of protein translation as primary mechanism to protect the individual against virus replication by preventing viral structural protein synthesis have been discussed in our previous report [19]. The mechanism including the protection against viral dsRNA via inhibition of protein synthesis is obviously a main immune response in cows vaccinated prior to parturition. This mechanism seems to be impaired in cows vaccinated in the period after calving, consequently, the AC cows are compromised regarding the ability to incapacitate virus replication and spread by activating the respective antiviral processes regulating the shut-off of protein synthesis.

#### Complex transcriptional upregulation of immune defense processes in cows vaccinated prior to calving

In the BC group, the large number of most significantly enriched canonical pathways indicates that a wide spectrum of signal cascades involved in immune defense is substantially modulated in response to antigen challenge (Table 3, S10 Table). The focus is on signaling mechanisms associated with immune response and anti-inflammatory actions, like cytokine signaling, JAK/stat signaling, glucocorticoid receptor signaling, NF-kB activation by viruses, B cell and T cell receptor signaling and PI3K/AKT signaling. Furthermore, several hormone signaling pathways were found to be enriched, like prolactin, estrogen receptor, glucocorticoid receptor and growth hormone signaling, which are involved in categories like reproduction, growth, differentiation and development and also have a close association to processes related to immune response. Furthermore, canonical pathways targeting cellular differentiation, development, growth, survival (mTOR signaling, FLT3 signaling in hematopoietic progenitor cells, erythropoietin and thrombopoietin signaling, IGF-1, GM-CSF, FAK, HGF, CNTF, VEGF, ERK5, rac and myc-mediated apoptosis signaling) as well as cellular pathways of adhesion, communication and migration (integrin signaling) were modulated at a highly significant magnitude. All of these pathways are represented by a variety of genes that followed a predominant trend of upregulation (Table 3, S10 Table) and suggest a comprehensive and coordinated immune reaction in the blood transcriptome of the BC cows for protection against antigen exposure, a transcriptional signature divergent to the AC cows.

#### Specific regulation of transcriptional immune defense response in cows vaccinated after calving

Compared to the BC cow group, the number and the variety of different canonical pathways involved in immunomodulation and maintenance of immnunocompetence are considerably lower in the blood transcriptome of AC cows (Table 4) highlighting again the reduced immune response in cows vaccinated after calving. However, a slight but coordinated upregulation of the initiation of the antibody-driven classical pathway of complement system cascade [43] was observed in the AC cows. The expression levels of the three complement 1q genes, *C1QA*, *C1QB* and *C1QC*, were moderately upregulated in response to antigen challenge (S1 Table). The activation of this canonical pathway, which is crucial for antibody mediated antigen clearance via the innate immune system, is also supported by the functional analyses of KEGG pathways (S9 Table). The C1Q loci play an important role for pattern recognition receptors in recognition of bacteria and viruses representing, a pathway upregulated in this group as well. In contrast, cows of the BC group did not show indication of the complement pathway modulation in response to vaccination.

In AC cows, the gene most significantly upregulated in response to vaccination was the unannotated, potentially cytokine-like locus *XLOC_32517* (S3 Table), which corresponds to the results from our previous analysis across all 12 cows included in the vaccination experiment [19]. The *XLOC_32517* locus that displays similarity regarding sequence and structural features to CSF2 (colony, or granulocyte macrophage, -stimulating factor 2) was also significantly upregulated in the BC group (S3 Table). Due to its cytokine similarity features it can be assumed that the XLOC_32517 locus might be involved in growth and differentiation of hematopoietic precursor cells from various lineages, including granulocytes, macrophages, eosinophils and erythrocytes, stimulation of which is of higher relevance for the response to antigen challenge in early lactating cows.

An example for a contrary transcriptional modulation in response to vaccination challenge in AC and BC cows is the locus *LOC444875* that is similar to immunoglobulin M (*IgM*). *LOC444875* is moderately downregulated in the AC cows (S1 Table) but upregulated in the BC cow group (S2 Table). IgM antibodies have been found to play an important role not only in primary defense mechanisms (transient immunity) but also in long-term protection against a variety of pathogens [44]. The contrary transcriptional modulation of the *LOC444875* locus in both cow groups, particularly the downregulation in the AC cows, supports the observation of an impaired immune system of cows in the early postpartal period.

**Fig 4:**
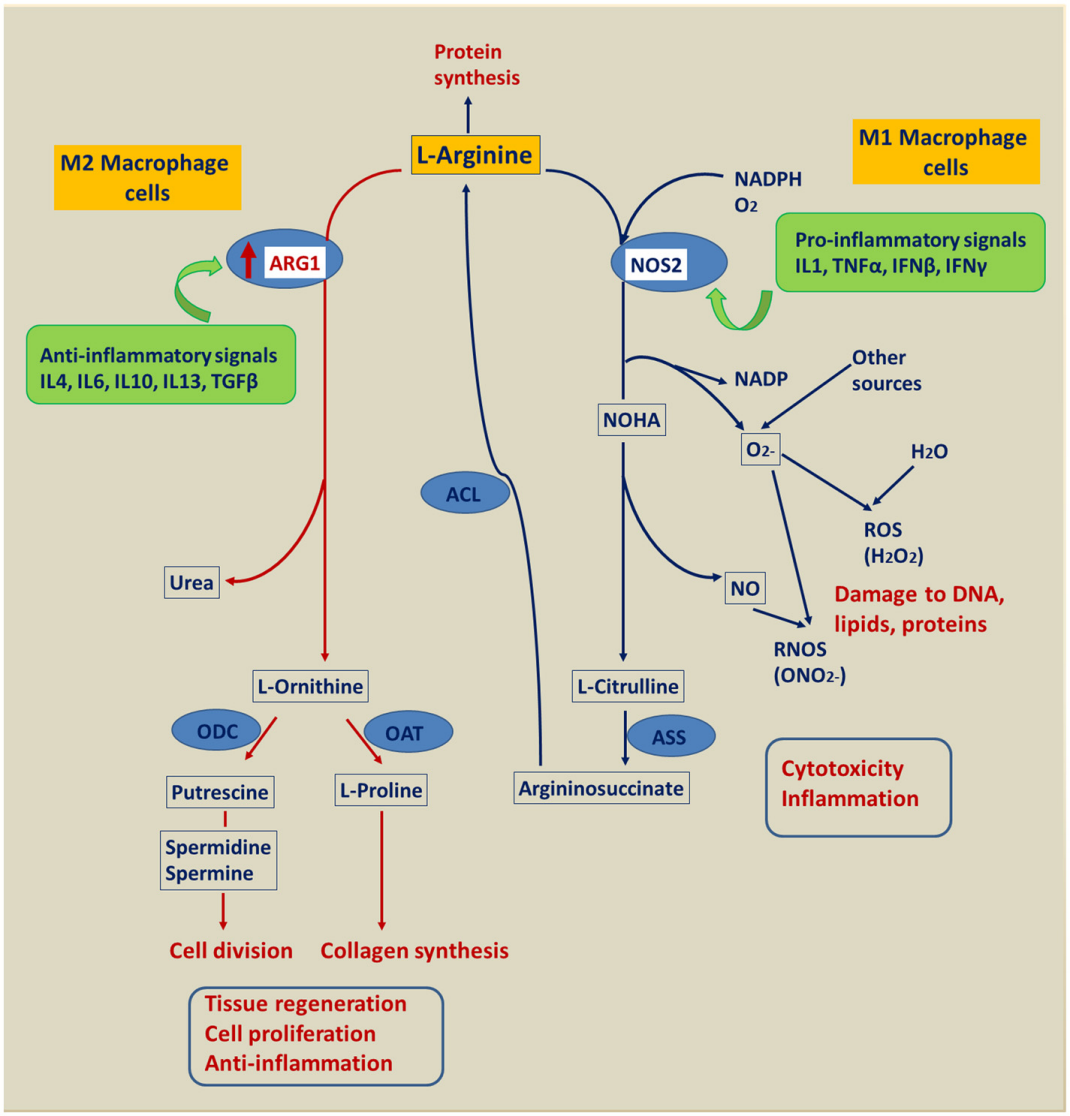
Model of reciprocal regulation of arginase 1 (ARG1) and nitric oxide synthase 2 (NOS2). Downstream metabolic products (blue framed) and their association with components of inflammatory responses (red). Enzymes (blue ellipses), NOHA: N-hydroxyarginine, OAT: ornithine aminotransferase, ODC: ornithine decarboxylase, ACL: argininosuccinate lyase, ASS: argininosuccinate synthetase. ROS: reactive oxygen species, RNOS: reactive nitrogen oxide species. Red arrows indicate consequences of ARG1 upregulation in AC cows vaccinated after calving.

A prominent difference in response to antigen challenge between both cow groups is the unique activation of the arginine degradation canonical pathway in the AC group (Table 4), which is supported by a highly significantly upregulated *ARG1* (arginase 1) expression (S1 Table). In contrast, the expression of *ARG1* was not affected in the BC cows. Stimulation of ARG1 activity reduces the availability of arginine as precursor for NO synthesis via inducible nitric oxide synthase (NOS2). A differential regulation pattern of NOS2 and ARG1 is thought to correspond to either classical (M1) or alternative (M2) macrophage activation, although this mechanism is still under debate and a more complex regulatory network might be responsible for macrophage polarization [45,46]. As displayed in Fig 4, ARG1 activity is switched on in M2 macrophages via IL10/IL4/IL13 induction. Activated ARG1 function has been reported to compensate unrestrained inflammation by producing cytokines suppressing the NO-mediated inflammation and cytotoxicity immune response. Thus, L-arginine metabolism in macrophages is a key determinant of defense against pathogen infection and has emerged as a defining feature of alternative versus classical macrophage activation [45–47]. It has been reported that M2 activated macrophages possess various functions including regulation of tissue remodeling processes, angiogenesis and antibody-mediated inflammatory responses [48–50]. In humans, myeloid cell ARG1-mediated L-arginine depletion was found to profoundly suppress T cell immune responses, and this has emerged as a fundamental mechanism of inflammation-associated immunosuppression [51,52]. Based on the results from the literature and from our vaccination experiment, the upregulation of *ARG1* expression in the blood of AC cows may point to an immunosuppressive mechanism underlying the impaired immune capacity of cows after calving. The ornithine generated by ARG1 action can be further metabolized to proline and polyamines, which are involved in cell growth and differentiation and collagen synthesis [53]. In addition, M2 macrophage activation is known to promote blood vessel formation, tissue repair and remodeling [46,54], which is generally necessary in the interval after calving.

#### Genes with a functional role in blood cell differentiation and development are upregulated in cows vaccinated after calving

**Fig 5:**
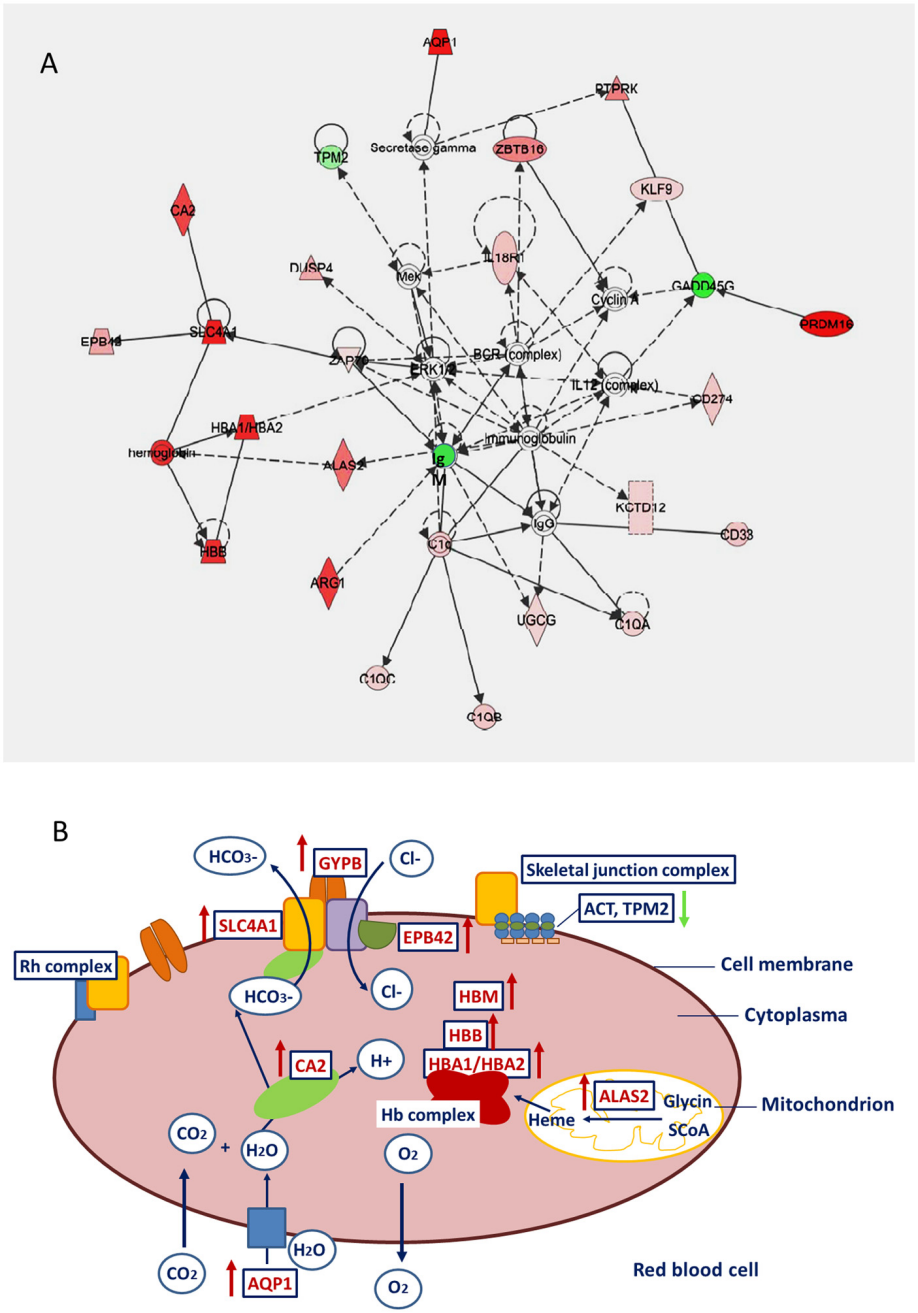
Vaccination-induced upregulation of erythrocyte-specific genes involved in gas exchange in early lactating cows. A: Interaction network for erythrocyte-enriched genes modulated in cows vaccinated after calving (AC) using IPA network analysis. Red: upregulated loci, green: downregulated loci. B: Model of the synergistic coactions of genes from the integrated gas exchange in the red blood cell. Genes (blue-framed boxes) upregulated in response to vaccination are indicated by red arrows. AQP1: aquaphorin, SLC4A1: band 3 anion exchange protein, CA2: carbonic anhydrase II, GYPB: glycophorin B, HBA, HBB: hemoglobin chains A and B, HBM: μ-globin, ALAS2: 5-aminolevulinate synthase 2, EPB42: erythrocyte membrane protein 4.2, ATPIF1: ATPase inhibitory factor 1, ACT: actin, TPM2: tropomyosin 2.

A signature specific for the transcriptional response in the blood of AC cows due to vaccination is the upregulation of tetrapyrrole biosynthesis pathway (Table 4) represented by increased expression of erythrocyte-specific *ALAS2* (5-aminolevulinate synthase 2, S1 Table) catalysing the rate-limiting step in heme biosynthesis from glycine in erythroid cells [55]. Out of the most significantly affected loci in the AC group after vaccination, the analysis revealed seven loci (S1 Table), which represent genes that operate synergistically in the major function of erythrocyte cells associated with respiratory gas exchange regulating the uptake of carbon dioxide and release of oxygen by erythrocytes: aquaphorin 1 (*AQP1*), solute carrier family 4, anion exchanger, member 1 (*SLC4A1*), carbonic anhydrase II (*CA2*), glycophorin B (*GYPB*), hemoglobin chains A and B (*HBA*, *HBB*) and μ-globin (*HBM*). The coordinated upregulation of the respective gene network (Fig 5A) was exclusively observed in the AC cows. The upregulation of the gas exchange is pathway in the AC cows is also supported by the functional enrichment analyses of the respective GO terms and KEGG pathways (S5 and S9 Tables). The synergistic modulation of the transcripts integrated in the gas exchange metabolon in the blood transcriptome of AC cows in response to vaccination is reflected in Fig 5B. The respective loci included in the gas exchange pathway are important for erythrocyte membrane biogenesis and responsible to maintain the erythrocyte shape and integrity [56–60] and their concerted upregulation also point to activated hematopoiesis and erythropoiesis processes. The primary function of erythrocytes is to transport oxygen in blood, and the primary stimulus for erythrocyte production is low oxygen levels. Enhanced oxygen delivery to oxygen-consuming tissues is required after calving and during early lactation.

Thus, the upregulation of genes involved in gas transport enables the AC cows to adjust for the increased demands for oxygen and energy required for regenerative processes to recover from blood loss due to calving, in the energy-consuming tissues at the onset of lactation (e.g., for lactogenesis in the mammary gland) and for the vaccination challenge on the immune system. Possibly, the transcriptional profile modulated in the blood of AC cows in response to vaccination might be compromised by the consequences arising out of blood loss and forcing the activation of red blood cell formation postpartum. However, the coordinated upregulation of the respective genes underlines that this process is of primary relevance and a specific transcriptional signature for the blood of this cow group.

#### Impaired respiratory chain pathway as immune response in cows vaccinated prior to calving

**Fig 6:**
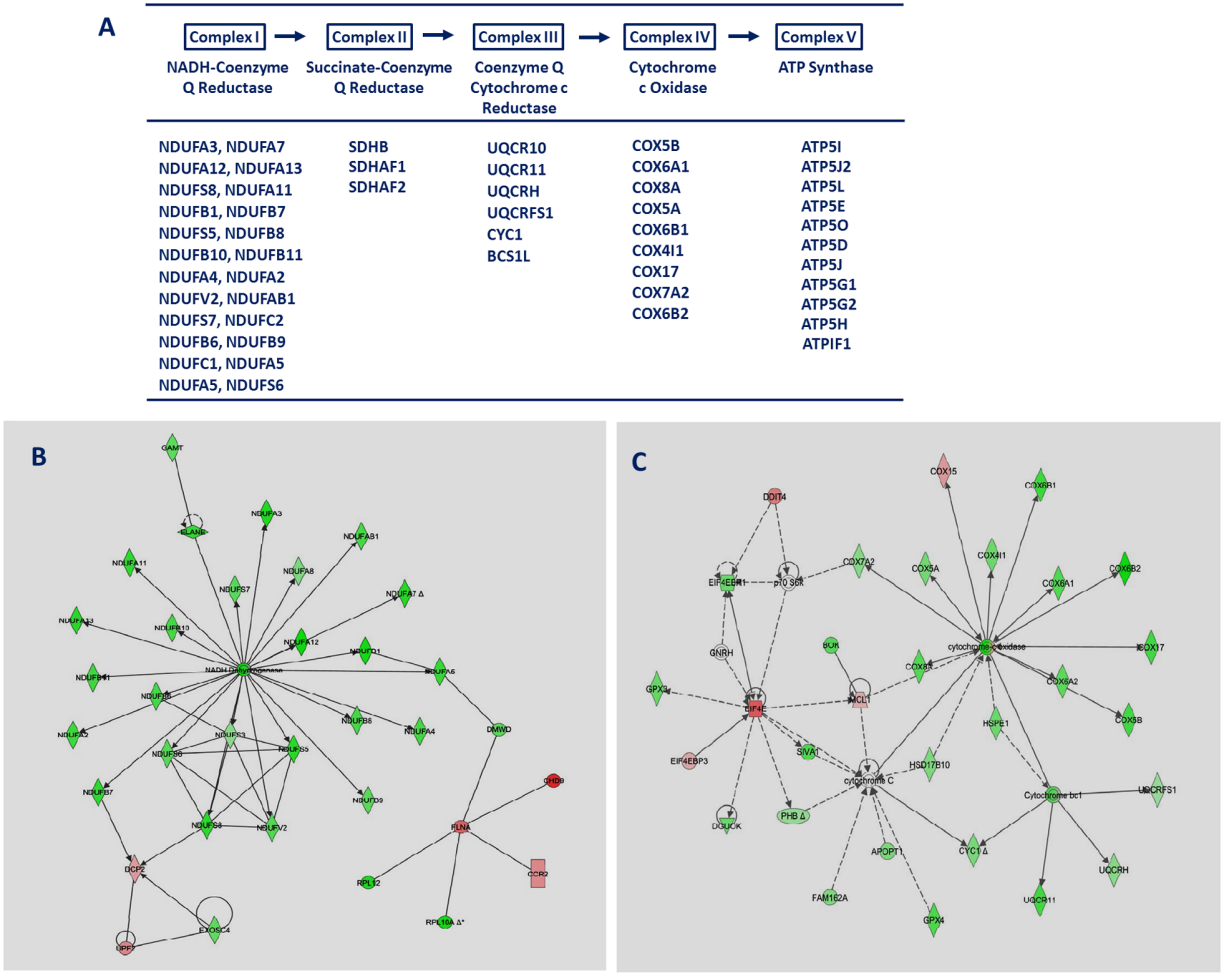
Interaction networks of modulated genes included in the electron transport chain in cows vaccinated prior to calving. A: Downregulated genes included in the electron transport chain in cows vaccinated prior to calving (BC). Red: upregulated loci, green: downregulated loci. B: Interaction networks for loci in complex I in BC cows using IPA network analysis. C: Interaction networks for loci in complex III and IV in BC cows using IPA network analysis. Red: upregulated loci, green: downregulated loci.

Strikingly, the canonical pathway of mitochondrial dysfunction was substantially affected after vaccination in the blood of BC cows. Out of the 53 modulated loci, which are involved in this pathway, 34% were downregulated and 4% were upregulated (Table 3, S10 Table). Downstream analysis showed that the majority of the affected loci are members of complex I of the electron transport chain (ETC), which catalyzes the transfer of electrons from NADH to ubiquinone in the inner mitochondrial membrane, but other ETC complexes were also modulated (Fig 6A–6C). The remarkable transcriptional downregulation of the genes involved in the mitochondrial ETC in response to vaccination was reflected by results of the functional enrichment analyses of KEGG pathways, which indicated that the oxidative phosphorylation (OXPHOS) pathway is significantly affected (S8 Table). Downregulation of a substantial number of genes critical for the mitochondrial function and OXPHOS system points to a repression of ATP production via this pathway in the blood of BC cows. Particularly, the structural integrity of the complex I assembly is known to be essential to maintain its functionality, and an impairment may lead to catalytic problems or instability of the complex assembly [61,62].

The mitochondrial ETC is also a major source for the generation of reactive oxygen species (ROS) due to direct electron leakage to oxygen at different redox steps of this process [62,63], which can have beneficial or detrimental effects. Excessive mitochondrial ROS is known to induce damages of cell components and leads to cell clearance through apoptosis, but ROS abundance is also an important intermediate signal for normal cell immune function, like control of T cell activation [64,65]. In addition to the impaired blood transcript levels of the loci involved in the ETC, an increased expression level was observed for *NNT* (nicotinamide nucleotide transhydrogenase) in response to vaccination in the BC cows. NNT functions as a redox-driven proton pump catalyzing the hydride transfer between NADH and NADP(+) for proton translocation across the inner mitochondrial membrane. NNT was reported to play a specific role in the control of ROS production and cellular redox state via NADPH [66], in regulating the cofactor balance by coordinating reductive carboxylation and glucose catabolism in the mitochondrial TCA cycle [67] and in modulating macrophage inflammatory responses. NNT overexpression was found to result in reduced intracellular ROS and NO levels and impaired intracellular bacteria clearance [68]. Control of intracellular ROS level is critical for cell survival. Modulation of transcripts associated with ROS production was not observed in BC cows, possibly due to complex I deficiency.

The collectively downregulated expression of loci involved in the ETC on one hand side and the increased *NNT* transcript abundance in blood of BC cows in response to vaccination on the other side suggest that mitochondria may play a role in regulating the immune response to vaccination in the BC cows. In contrast, those vaccination-evoked effects were not observed in the blood transcriptome of AC cows. Mitochondria have the capacity to sense energy status of the cell as well as a variety of cellular stresses and respond with initiation of cell death. Reviewing recent studies about mitochondrial function in pathogen recognition, Tal and Iwasaki [69] concluded that the mitochondria are shaping the innate response to pathogens, and they suggest the existence of a “mitoxosome”, a mitochondrial signalosome or integral platform, where multiple pathways of viral recognition and cellular stress signals converge and are coordinated to generate an antiviral response.

#### Shifted blood cell energy metabolism towards glycolysis in cows vaccinated prior to calving

The impairment of the ETC pathway observed as a specific transcriptional signature in the blood of BC in response to vaccination (see previous section) implies that the subsequently reduced ATP generation via TCA cycle might be simultaneously linked with a reprogramming of energy metabolism to rebalance ATP production. The *PDK4* (pyruvate dehydrogenase kinase, isozyme 4) transcript abundance increased in parallel in the blood of BC cows in response to vaccination (S2 Table), which fits in the hypothesis of reprogrammed ATP production. PDK4 is known to play a crucial role in glucose utilization and lipid metabolism by regulating the mitochondrial pyruvate dehydrogenase (PDH) complex that converts pyruvate to acetyl CoA for oxidation and ATP generation in the TCA cycle. Elevated PDK4 expression leads to a suppression of the influx of glycolytic metabolites into mitochondria [70], which in turn, would support the reduction of ATP production via TCA cycle. In cancer cells, a low level of glucose oxidation via TCA cycle has been shown to be due to the constant inhibition of PDH by phosphorylation of one of its four kinases [71–73]. Recently, PDK4 was identified as a novel regulator of one of the signaling pathways of mTORC1 via HIFA1 effects and of aerobic glycolysis [74] as well as a novel potential hub that may play a central role in the complex interplay of transcription-factors, transcripts and lipids during the course of monocyte-macrophage differentiation [75].

It has been reported that upon immune challenge and during the adaptive immune response, lymphocytes undergo rapid dramatic metabolic changes due to reprogramming from a quiescent cell status to an activated one. This process is characterized by a switch from the ATP production via TCA cycle to the diametrical aerobic glycolysis pathway in order to adjust for high energy and metabolic demands required for cell proliferation, differentiation and cytokine production (e.g., [76–79]). This metabolic switch, a phenomenon known as the Warburg effect [80], is accepted as a metabolic signature of highly proliferating cells, not only cancer cells but also activated lymphocytes [79,81,82], TLR-activated and NO-producing inflammatory dendritic cells [83–85] as a survival response that serves to rebalance ATP levels and to rapidly provide glycolytic intermediates for biosynthetic growth and proliferation processes. Although ATP production via OXPHOS is more efficient than via glycolysis, for macromolecule synthesis and proliferation, glycolysis has been reported to produce ATP at a rate 100 times faster than OXPHOS [86].

**Fig 7:**
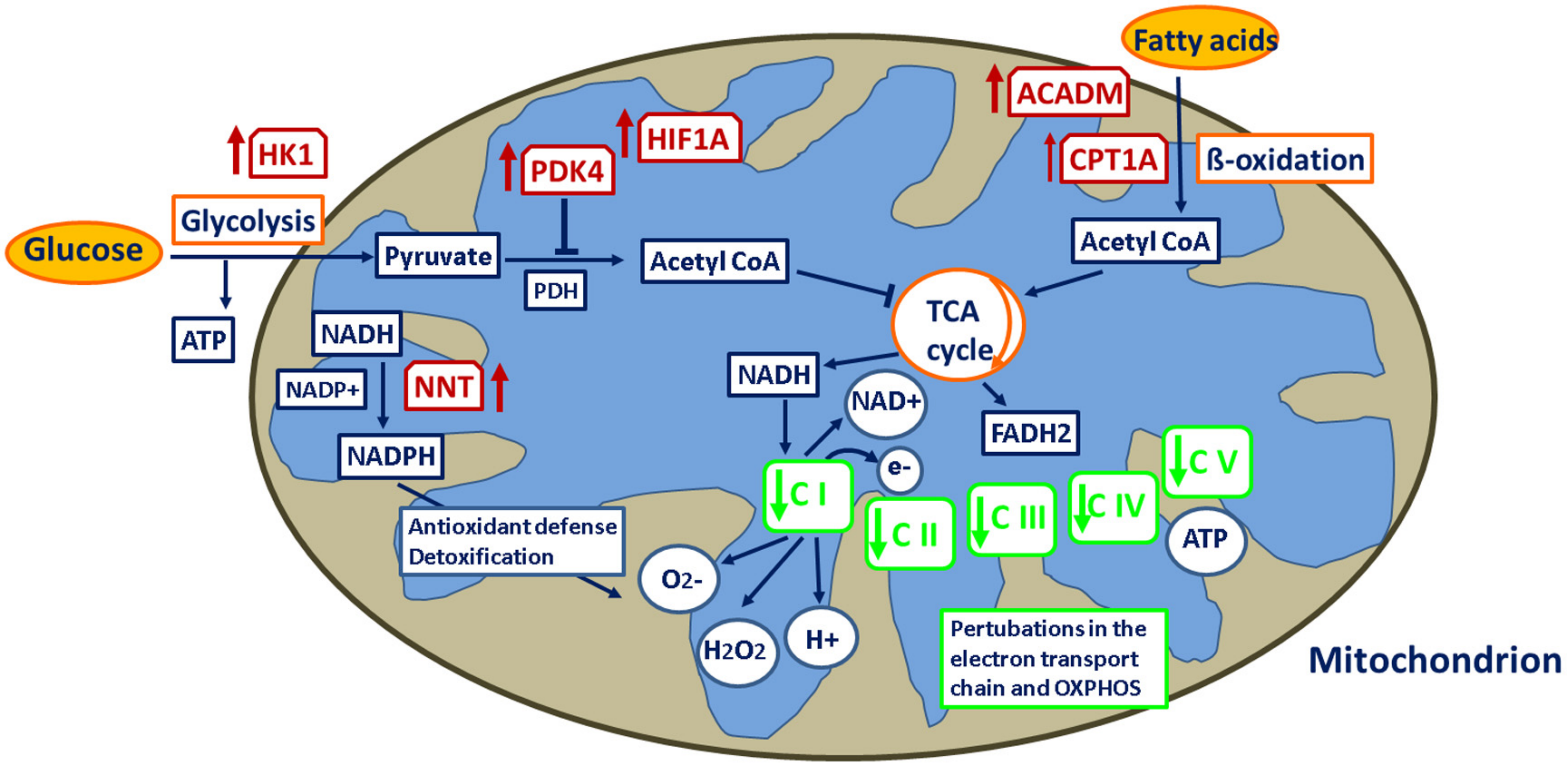
Metabolic switch due to virus challenge in cows vaccinated prior to calving. Loci (red-framed boxes) upregulated in response to vaccination are indicated by red arrows, loci (green-framed boxes) downregulated in response to vaccination are indicated by green arrows. CI-C V: complexes of the respiratory electron transport chain. PDK4: pyruvate dehydrogenase kinase 4, NNT: nicotinamide nucleotide transhydrogenase, CPT1A: carnitine palmitoyltransferase 1A, ACADM: acyl-CoA dehydrogenase, medium-chain, HK1: hexokinase 1, HIF1A: hypoxia inducible factor 1 alpha.

In our study, rebalancing of ATP generation via glycolysis is supported by expression upregulation of *HK1* (hexokinase 1) and transcription factor *HIF1A* (hypoxia inducible factor 1 alpha) in BC cows. HK1 phosphorylates glucose to produce glucose-6-phosphate, the first step in glycolysis, and HIF1A is a transcriptional factor known to control metabolism, survival and innate immunity in response to inflammation and oxygen deficiency [87] as well as to regulate glycolysis stimulation [88] and cellular reprogramming [89]. The vaccination-induced mitochondrial deficiency and the upregulation of PDK4 and HIF1A may synergize to activate glycolysis to generate maximal quantities of ATP and metabolites required for blood/immune cell proliferation in response to antigen challenge (Fig 7). These regulatory metabolic mechanisms were not modulated in AC cows in response to vaccination. In early lactating AC cows, glucose is primarily needed for lactogenesis, thus availability of glucose for activation of lymphocytes is limited. This may lead to an inhibition of cell proliferation and cytokine production contributing to immunosuppression of these cows.

## Conclusions

In summary, the results of our study indicate a differential modulation of transcripts, biological processes and pathways in the two cow groups vaccinated prior to or after calving and show that the different physiological and metabolic status of the cows (non-lactating vs. early lactating) is clearly reflected at the whole transcriptome level in the blood of the divergent cow groups. The response to vaccination elicits a quantitatively and qualitatively divergent regulatory immune defense reaction in the blood transcriptome of each cow group. Remarkably, the non-lactating cows vaccinated prior to calving displayed a substantially higher number of differentially expressed loci compared to the early lactating cows vaccinated after calving. The lower magnitude of the overall transcriptional modulation in the early lactating cows suggests a generally attenuated reactivity in response to antigen challenge. The immune response of non-lactating cows involved an activation of a complex and divergent spectrum of signaling and metabolic pathways for protection against antigen challenge compared to the early lactating cows. Their immune response seemed to be compromised by temporary pivotal physiological challenges associated with the consequences due to calving and the onset of lactation. Thus, the unique vaccination-induced transcriptome signature in the blood of the early lactating cows is characterized by an impaired transcriptional regulation of specific loci associated with the immune defense response in non-lactating cows, which provides evidence for the suppressed capacity for immunomodulation on the molecular level in early lactating cows. The results of our study can contribute to build a model of key signaling pathways that are modulated divergently in response to vaccination and to identify potential specific biomarkers characteristic for divergent immune responses.

## Supporting Information

S1 Table Differentially expressed loci in response to vaccination in cows vaccinated after calving (AC). Gene_ID: official locus name or previously unknown locus (NA). LogFC: log fold change between expression levels prior and after vaccination. Significance threshold: q<0.05.
(XLSX)

S2 Table Differentially expressed loci in response to vaccination in cows vaccinated prior to calving (BC). Gene_ID: official locus name or previously unknown locus (NA). LogFC: log fold change between expression levels prior and after vaccination. Significance threshold: q<0.05.
(XLSX)

S3 Table Differentially expressed loci in response to vaccination in both cow groups (overlap AC+BC). Gene_ID: official locus name or previously unknown locus (NA). LogFC: log fold change between expression levels prior to and after vaccination provided for cow groups AC and BC respectively. Significance threshold: q<0.05.
(XLSX)

S4 Table Significantly enriched GO terms of differentially expressed genes in response to vaccination of cows prior to calving. Significance threshold: q<0.05.
(DOCX)

S5 Table Significantly enriched GO terms of differentially expressed genes in response to vaccination of cows after calving. Significance threshold: q<0.05.
(DOCX)

S6 Table Biological functions (IPA categories) most significantly modulated by differentially expressed genes in response to vaccination prior to calving (BC). The column “categories biological function” summarises the affected biological functions into categories according to IPA analysis. The range of p values for overrepresentation of single biological functions (p-value_min, p-value_max) is indicated. The predicted activation status (z-score) and the number of differentially expressed genes (# molecules: maximal number of molecules affected) assigned to the respective function are indicated.
(XLSX)

S7 Table Biological functions (IPA categories) most significantly modulated by differentially expressed genes in response to vaccination after calving (AC). The column “categories biological function” summarises the affected biological functions into categories according to IPA analysis. The range of p values for overrepresentation of single biological functions (p-value_min, p-value_max) is indicated. The predicted activation status (z-score) and the number of differentially expressed genes (# molecules: maximal number of molecules affected) assigned to the respective function are indicated.
(XLSX)

S8 Table Significantly affected KEGG pathways of differentially expressed genes in response to vaccination prior to calving. Significance threshold: q<0.05.
(DOCX)

S9 Table Significantly affected KEGG pathways of differentially expressed genes in response to vaccination after calving. Significance threshold: q<0.05.
(DOCX)

S10 Table Canonical pathways significantly enriched by differentially expressed genes in response to vaccination prior to calving. The ratio provides the proportion of genes in the respective canonical pathway, which are significantly differentially expressed after vaccination, relative to all genes present in the pathway. The proportion of upregulated and downregulated genes in the respective pathway and a full list of all differentially expressed genes assigned to the respective pathway are indicated. Significance threshold: p<0.05.
(XLSX)
