# Supplementary material for: Different Blood Cell-Derived Transcriptome Signatures in Cows Exposed to Vaccination Pre- or Postpartum
Source: PLoS One. 2015 Aug 28;10(8):e0136927. doi: 10.1371/journal.pone.0136927 (PMC4552870; doi:10.1371/journal.pone.0136927)
Supplement: S4 Table — Significance threshold: q<0.05. (DOCX) [file pone.0136927.s004.docx]

Additional file 4, Table S4: GO term enrichment in differentially-expressed genes in response to vaccination after calving (FDR, q< 0.05)

| **GO Category** | **Over represented (p-value)** | **q-value** | **GO Term**  **Biological process** |
| --- | --- | --- | --- |
| GO:0006415 | 1,79E-39 | 2,63E-35 | translational termination |
| GO:0006613 | 1,66E-37 | 1,22E-33 | cotranslational protein targeting to membrane |
| GO:0006614 | 7,56E-37 | 2,78E-33 | SRP-dependent cotranslational protein targeting to membrane |
| GO:0022626 | 7,18E-37 | 2,78E-33 | cytosolic ribosome |
| GO:0045047 | 2,05E-36 | 5,02E-33 | protein targeting to ER |
| GO:0072599 | 2,05E-36 | 5,02E-33 | establishment of protein localization to endoplasmic reticulum |
| GO:0003735 | 5,08E-36 | 1,07E-32 | structural constituent of ribosome |
| GO:0006413 | 7,52E-35 | 1,38E-31 | translational initiation |
| GO:0044445 | 1,65E-34 | 2,70E-31 | cytosolic part |
| GO:0044391 | 1,35E-33 | 1,98E-30 | ribosomal subunit |
| GO:0006414 | 4,19E-33 | 5,60E-30 | translational elongation |
| GO:0000184 | 3,05E-32 | 3,45E-29 | nuclear-transcribed mRNA catabolic process, nonsense-mediated decay |
| GO:0034623 | 3,03E-32 | 3,45E-29 | cellular macromolecular complex disassembly |
| GO:0070972 | 3,81E-32 | 4,00E-29 | protein localization to endoplasmic reticulum |
| GO:0000956 | 4,82E-32 | 4,72E-29 | nuclear-transcribed mRNA catabolic process |
| GO:0032984 | 5,91E-32 | 5,42E-29 | macromolecular complex disassembly |
| GO:0043624 | 1,53E-31 | 1,33E-28 | cellular protein complex disassembly |
| GO:0005840 | 2,32E-31 | 1,89E-28 | ribosome |
| GO:0043241 | 3,46E-31 | 2,67E-28 | protein complex disassembly |
| GO:0032991 | 1,08E-29 | 7,90E-27 | macromolecular complex |
| GO:0006402 | 2,47E-29 | 1,73E-26 | mRNA catabolic process |
| GO:0006612 | 2,77E-27 | 1,85E-24 | protein targeting to membrane |
| GO:0006401 | 3,77E-27 | 2,41E-24 | RNA catabolic process |
| GO:0019080 | 4,52E-27 | 2,66E-24 | viral genome expression |
| GO:0019083 | 4,52E-27 | 2,66E-24 | viral transcription |
| GO:0022411 | 5,58E-25 | 3,15E-22 | cellular component disassembly |
| GO:0071845 | 7,11E-25 | 3,87E-22 | cellular component disassembly at cellular level |
| GO:0019058 | 1,46E-24 | 7,65E-22 | viral infectious cycle |
| GO:0072594 | 3,33E-23 | 1,69E-20 | establishment of protein localization to organelle |
| GO:0022627 | 1,05E-22 | 5,14E-20 | cytosolic small ribosomal subunit |
| GO:0006412 | 1,52E-22 | 7,19E-20 | translation |
| GO:0016032 | 1,97E-22 | 9,03E-20 | viral reproduction |
| GO:0030529 | 4,35E-22 | 1,94E-19 | ribonucleoprotein complex |
| GO:0022904 | 2,77E-21 | 1,20E-18 | respiratory electron transport chain |
| GO:0022415 | 1,35E-20 | 5,65E-18 | viral reproductive process |
| GO:0005198 | 8,99E-20 | 3,67E-17 | structural molecule activity |
| GO:0044265 | 7,38E-19 | 2,93E-16 | cellular macromolecule catabolic process |
| GO:0034621 | 1,15E-18 | 4,43E-16 | cellular macromolecular complex subunit organization |
| GO:0015935 | 5,14E-18 | 1,93E-15 | small ribosomal subunit |
| GO:0015934 | 1,75E-17 | 6,44E-15 | large ribosomal subunit |
| GO:0016071 | 1,99E-17 | 7,15E-15 | mRNA metabolic process |
| GO:0044455 | 3,37E-17 | 1,18E-14 | mitochondrial membrane part |
| GO:0044424 | 4,45E-17 | 1,52E-14 | intracellular part |
| GO:0033365 | 1,00E-16 | 3,35E-14 | protein localization to organelle |
| GO:0070469 | 3,09E-16 | 1,01E-13 | respiratory chain |
| GO:0022900 | 4,56E-16 | 1,46E-13 | electron transport chain |
| GO:0005622 | 5,73E-16 | 1,79E-13 | intracellular |
| GO:0022625 | 6,30E-16 | 1,93E-13 | cytosolic large ribosomal subunit |
| GO:0005829 | 8,76E-16 | 2,63E-13 | cytosol |
| GO:0009057 | 2,15E-15 | 6,32E-13 | macromolecule catabolic process |
| GO:0005747 | 2,72E-15 | 7,55E-13 | mitochondrial respiratory chain complex I |
| GO:0030964 | 2,72E-15 | 7,55E-13 | NADH dehydrogenase complex |
| GO:0045271 | 2,72E-15 | 7,55E-13 | respiratory chain complex I |
| GO:0048610 | 3,01E-15 | 8,18E-13 | cellular process involved in reproduction |
| GO:0043229 | 3,54E-15 | 9,45E-13 | intracellular organelle |
| GO:0071842 | 4,35E-15 | 1,14E-12 | cellular component organization at cellular level |
| GO:0043226 | 4,54E-15 | 1,17E-12 | organelle |
| GO:0044444 | 5,23E-15 | 1,33E-12 | cytoplasmic part |
| GO:0009059 | 5,63E-15 | 1,40E-12 | macromolecule biosynthetic process |
| GO:0010467 | 6,01E-15 | 1,43E-12 | gene expression |
| GO:0044446 | 6,05E-15 | 1,43E-12 | intracellular organelle part |
| GO:0071841 | 6,05E-15 | 1,43E-12 | cellular component organization or biogenesis at cellular level |
| GO:0005746 | 7,95E-15 | 1,85E-12 | mitochondrial respiratory chain |
| GO:0044422 | 8,22E-15 | 1,89E-12 | organelle part |
| GO:0046907 | 1,63E-14 | 3,68E-12 | intracellular transport |
| GO:0003954 | 4,92E-14 | 1,06E-11 | NADH dehydrogenase activity |
| GO:0008137 | 4,92E-14 | 1,06E-11 | NADH dehydrogenase (ubiquinone) activity |
| GO:0050136 | 4,92E-14 | 1,06E-11 | NADH dehydrogenase (quinone) activity |
| GO:0034645 | 5,01E-14 | 1,07E-11 | cellular macromolecule biosynthetic process |
| GO:0006605 | 5,92E-14 | 1,24E-11 | protein targeting |
| GO:0045333 | 1,39E-13 | 2,88E-11 | cellular respiration |
| GO:0043234 | 2,02E-13 | 4,12E-11 | protein complex |
| GO:0006120 | 2,05E-13 | 4,13E-11 | mitochondrial electron transport, NADH to ubiquinone |
| GO:0071822 | 3,41E-13 | 6,76E-11 | protein complex subunit organization |
| GO:0044249 | 5,59E-13 | 1,09E-10 | cellular biosynthetic process |
| GO:0044267 | 5,73E-13 | 1,11E-10 | cellular protein metabolic process |
| GO:0042773 | 6,88E-13 | 1,30E-10 | ATP synthesis coupled electron transport |
| GO:0042775 | 6,88E-13 | 1,30E-10 | mitochondrial ATP synthesis coupled electron transport |
| GO:0016043 | 7,09E-13 | 1,32E-10 | cellular component organization |
| GO:0051649 | 8,36E-13 | 1,53E-10 | establishment of localization in cell |
| GO:0044237 | 8,71E-13 | 1,58E-10 | cellular metabolic process |
| GO:0071840 | 9,36E-13 | 1,68E-10 | cellular component organization or biogenesis |
| GO:0006886 | 1,13E-12 | 2,00E-10 | intracellular protein transport |
| GO:0043933 | 1,23E-12 | 2,15E-10 | macromolecular complex subunit organization |
| GO:0003723 | 1,91E-12 | 3,30E-10 | RNA binding |
| GO:0044260 | 1,96E-12 | 3,34E-10 | cellular macromolecule metabolic process |
| GO:0044248 | 2,44E-12 | 4,11E-10 | cellular catabolic process |
| GO:0051641 | 3,26E-12 | 5,44E-10 | cellular localization |
| GO:0009058 | 3,76E-12 | 6,21E-10 | biosynthetic process |
| GO:0019866 | 6,71E-12 | 1,10E-09 | organelle inner membrane |
| GO:0033036 | 1,13E-11 | 1,82E-09 | macromolecule localization |
| GO:0006139 | 2,12E-11 | 3,38E-09 | nucleobase-containing compound metabolic process |
| GO:0006119 | 2,34E-11 | 3,70E-09 | oxidative phosphorylation |
| GO:0005737 | 2,96E-11 | 4,62E-09 | cytoplasm |
| GO:0043170 | 3,61E-11 | 5,58E-09 | macromolecule metabolic process |
| GO:0005743 | 4,37E-11 | 6,69E-09 | mitochondrial inner membrane |
| GO:0005623 | 4,57E-11 | 6,85E-09 | cell |
| GO:0044464 | 4,57E-11 | 6,85E-09 | cell part |
| GO:0008104 | 5,08E-11 | 7,54E-09 | protein localization |
| GO:0043228 | 5,56E-11 | 8,09E-09 | non-membrane-bounded organelle |
| GO:0043232 | 5,56E-11 | 8,09E-09 | intracellular non-membrane-bounded organelle |
| GO:0034613 | 5,97E-11 | 8,60E-09 | cellular protein localization |
| GO:0016655 | 6,04E-11 | 8,61E-09 | oxidoreductase activity, acting on NADH or NADPH, quinone or similar compound as acceptor |
| GO:0016070 | 6,74E-11 | 9,52E-09 | RNA metabolic process |
| GO:0043227 | 7,79E-11 | 1,09E-08 | membrane-bounded organelle |
| GO:0043231 | 7,99E-11 | 1,11E-08 | intracellular membrane-bounded organelle |
| GO:0019843 | 8,37E-11 | 1,14E-08 | rRNA binding |
| GO:0070727 | 8,37E-11 | 1,14E-08 | cellular macromolecule localization |
| GO:0022414 | 9,85E-11 | 1,33E-08 | reproductive process |
| GO:0019538 | 1,08E-10 | 1,44E-08 | protein metabolic process |
| GO:0015031 | 1,28E-10 | 1,69E-08 | protein transport |
| GO:0000003 | 1,37E-10 | 1,80E-08 | reproduction |
| GO:0034641 | 1,82E-10 | 2,36E-08 | cellular nitrogen compound metabolic process |
| GO:0045184 | 2,67E-10 | 3,44E-08 | establishment of protein localization |
| GO:0008152 | 3,57E-10 | 4,55E-08 | metabolic process |
| GO:0006807 | 3,93E-10 | 4,98E-08 | nitrogen compound metabolic process |
| GO:0032774 | 1,31E-09 | 1,65E-07 | RNA biosynthetic process |
| GO:0015980 | 3,20E-09 | 3,99E-07 | energy derivation by oxidation of organic compounds |
| GO:0090304 | 3,33E-09 | 4,11E-07 | nucleic acid metabolic process |
| GO:0009056 | 3,76E-09 | 4,56E-07 | catabolic process |
| GO:0016651 | 3,75E-09 | 4,56E-07 | oxidoreductase activity, acting on NADH or NADPH |
| GO:0009987 | 7,27E-09 | 8,75E-07 | cellular process |
| GO:0005515 | 1,09E-08 | 1,30E-06 | protein binding |
| GO:0048535 | 1,90E-08 | 2,26E-06 | lymph node development |
| GO:0005740 | 3,07E-08 | 3,61E-06 | mitochondrial envelope |
| GO:0006996 | 3,11E-08 | 3,63E-06 | organelle organization |
| GO:0044429 | 3,28E-08 | 3,80E-06 | mitochondrial part |
| GO:0031966 | 3,46E-08 | 3,97E-06 | mitochondrial membrane |
| GO:0031323 | 4,89E-08 | 5,57E-06 | regulation of cellular metabolic process |
| GO:0010604 | 4,96E-08 | 5,61E-06 | positive regulation of macromolecule metabolic process |
| GO:0031975 | 1,12E-07 | 1,26E-05 | envelope |
| GO:0031967 | 1,27E-07 | 1,41E-05 | organelle envelope |
| GO:0003676 | 1,35E-07 | 1,49E-05 | nucleic acid binding |
| GO:0080090 | 1,38E-07 | 1,51E-05 | regulation of primary metabolic process |
| GO:0031325 | 1,40E-07 | 1,53E-05 | positive regulation of cellular metabolic process |
| GO:0009893 | 1,70E-07 | 1,83E-05 | positive regulation of metabolic process |
| GO:0044238 | 2,16E-07 | 2,32E-05 | primary metabolic process |
| GO:0016050 | 2,70E-07 | 2,87E-05 | vesicle organization |
| GO:0008150 | 3,40E-07 | 3,59E-05 | biological_process |
| GO:0006091 | 9,65E-07 | 0,0001 | generation of precursor metabolites and energy |
| GO:0031981 | 9,72E-07 | 0,0001 | nuclear lumen |
| GO:0004298 | 1,16E-06 | 0,0001 | threonine-type endopeptidase activity |
| GO:0005839 | 1,16E-06 | 0,0001 | proteasome core complex |
| GO:0006901 | 1,16E-06 | 0,0001 | vesicle coating |
| GO:0031974 | 1,14E-06 | 0,0001 | membrane-enclosed lumen |
| GO:0070003 | 1,16E-06 | 0,0001 | threonine-type peptidase activity |
| GO:0060255 | 1,57E-06 | 0,0002 | regulation of macromolecule metabolic process |
| GO:0005164 | 2,03E-06 | 0,0002 | tumor necrosis factor receptor binding |
| GO:0044428 | 2,27E-06 | 0,0002 | nuclear part |
| GO:0002876 | 2,43E-06 | 0,0002 | positive regulation of chronic inflammatory response to antigenic stimulus |
| GO:0005575 | 2,95E-06 | 0,0003 | cellular_component |
| GO:0005753 | 3,00E-06 | 0,0003 | mitochondrial proton-transporting ATP synthase complex |
| GO:0006900 | 3,47E-06 | 0,0003 | membrane budding |
| GO:0032813 | 3,95E-06 | 0,0004 | tumor necrosis factor receptor superfamily binding |
| GO:0015078 | 4,57E-06 | 0,0004 | hydrogen ion transmembrane transporter activity |
| GO:0015985 | 5,14E-06 | 0,0005 | energy coupled proton transport, down electrochemical gradient |
| GO:0015986 | 5,14E-06 | 0,0005 | ATP synthesis coupled proton transport |
| GO:0043233 | 5,45E-06 | 0,0005 | organelle lumen |
| GO:0005654 | 5,96E-06 | 0,0006 | nucleoplasm |
| GO:0002437 | 6,75E-06 | 0,0006 | inflammatory response to antigenic stimulus |
| GO:0045259 | 7,29E-06 | 0,0007 | proton-transporting ATP synthase complex |
| GO:0044419 | 7,91E-06 | 0,0007 | interspecies interaction between organisms |
| GO:0000989 | 8,06E-06 | 0,0007 | transcription factor binding transcription factor activity |
| GO:0002676 | 8,41E-06 | 0,0008 | regulation of chronic inflammatory response |
| GO:0003712 | 9,21E-06 | 0,0008 | transcription cofactor activity |
| GO:0000988 | 1,13E-05 | 0,0010 | protein binding transcription factor activity |
| GO:0005739 | 1,13E-05 | 0,0010 | mitochondrion |
| GO:0002874 | 1,68E-05 | 0,0015 | regulation of chronic inflammatory response to antigenic stimulus |
| GO:0051171 | 1,78E-05 | 0,0015 | regulation of nitrogen compound metabolic process |
| GO:0006810 | 1,89E-05 | 0,0016 | transport |
| GO:0005634 | 2,06E-05 | 0,0018 | nucleus |
| GO:0042776 | 2,35E-05 | 0,0020 | mitochondrial ATP synthesis coupled proton transport |
| GO:0070013 | 2,60E-05 | 0,0022 | intracellular organelle lumen |
| GO:0019222 | 2,67E-05 | 0,0023 | regulation of metabolic process |
| GO:0009889 | 2,99E-05 | 0,0025 | regulation of biosynthetic process |
| GO:0042274 | 3,02E-05 | 0,0025 | ribosomal small subunit biogenesis |
| GO:0051234 | 3,15E-05 | 0,0026 | establishment of localization |
| GO:0006366 | 3,31E-05 | 0,0027 | transcription from RNA polymerase II promoter |
| GO:0009891 | 3,60E-05 | 0,0030 | positive regulation of biosynthetic process |
| GO:0048522 | 3,78E-05 | 0,0031 | positive regulation of cellular process |
| GO:0016310 | 4,01E-05 | 0,0033 | phosphorylation |
| GO:0019882 | 4,16E-05 | 0,0034 | antigen processing and presentation |
| GO:0002474 | 4,22E-05 | 0,0034 | antigen processing and presentation of peptide antigen via MHC class I |
| GO:0031326 | 4,23E-05 | 0,0034 | regulation of cellular biosynthetic process |
| GO:0009206 | 4,34E-05 | 0,0034 | purine ribonucleoside triphosphate biosynthetic process |
| GO:0043085 | 5,12E-05 | 0,0040 | positive regulation of catalytic activity |
| GO:0010557 | 5,56E-05 | 0,0044 | positive regulation of macromolecule biosynthetic process |
| GO:0009145 | 6,12E-05 | 0,0048 | purine nucleoside triphosphate biosynthetic process |
| GO:0002439 | 6,52E-05 | 0,0050 | chronic inflammatory response to antigenic stimulus |
| GO:0002678 | 6,52E-05 | 0,0050 | positive regulation of chronic inflammatory response |
| GO:0009152 | 6,51E-05 | 0,0050 | purine ribonucleotide biosynthetic process |
| GO:0017070 | 6,52E-05 | 0,0050 | U6 snRNA binding |
| GO:0045263 | 6,52E-05 | 0,0050 | proton-transporting ATP synthase complex, coupling factor F(o) |
| GO:0051179 | 7,26E-05 | 0,0055 | localization |
| GO:0019219 | 8,41E-05 | 0,0063 | regulation of nucleobase-containing compound metabolic process |
| GO:0002705 | 8,84E-05 | 0,0066 | positive regulation of leukocyte mediated immunity |
| GO:0002708 | 8,84E-05 | 0,0066 | positive regulation of lymphocyte mediated immunity |
| GO:0031328 | 9,42E-05 | 0,0070 | positive regulation of cellular biosynthetic process |
| GO:0000502 | 1,02E-04 | 0,0076 | proteasome complex |
| GO:0042090 | 1,04E-04 | 0,0076 | interleukin-12 biosynthetic process |
| GO:0045075 | 1,04E-04 | 0,0076 | regulation of interleukin-12 biosynthetic process |
| GO:0045084 | 1,04E-04 | 0,0076 | positive regulation of interleukin-12 biosynthetic process |
| GO:0010556 | 1,05E-04 | 0,0076 | regulation of macromolecule biosynthetic process |
| GO:0016192 | 1,11E-04 | 0,0080 | vesicle-mediated transport |
| GO:0002544 | 1,22E-04 | 0,0086 | chronic inflammatory response |
| GO:0002863 | 1,21E-04 | 0,0086 | positive regulation of inflammatory response to antigenic stimulus |
| GO:0033177 | 1,22E-04 | 0,0086 | proton-transporting two-sector ATPase complex, proton-transporting domain |
| GO:0051704 | 1,24E-04 | 0,0087 | multi-organism process |
| GO:0006338 | 1,55E-04 | 0,0109 | chromatin remodeling |
| GO:0009142 | 1,59E-04 | 0,0111 | nucleoside triphosphate biosynthetic process |
| GO:0009201 | 1,60E-04 | 0,0111 | ribonucleoside triphosphate biosynthetic process |
| GO:0051443 | 1,65E-04 | 0,0114 | positive regulation of ubiquitin-protein ligase activity |
| GO:0003713 | 1,71E-04 | 0,0118 | transcription coactivator activity |
| GO:0002923 | 1,88E-04 | 0,0128 | regulation of humoral immune response mediated by circulating immunoglobulin |
| GO:0002925 | 1,88E-04 | 0,0128 | positive regulation of humoral immune response mediated by circulating immunoglobulin |
| GO:0051427 | 1,88E-04 | 0,0128 | hormone receptor binding |
| GO:0032268 | 1,93E-04 | 0,0131 | regulation of cellular protein metabolic process |
| GO:0044093 | 2,14E-04 | 0,0145 | positive regulation of molecular function |
| GO:0043565 | 2,18E-04 | 0,0146 | sequence-specific DNA binding |
| GO:0002376 | 2,21E-04 | 0,0147 | immune system process |
| GO:0045954 | 2,21E-04 | 0,0147 | positive regulation of natural killer cell mediated cytotoxicity |
| GO:0009260 | 2,25E-04 | 0,0149 | ribonucleotide biosynthetic process |
| GO:0002922 | 2,33E-04 | 0,0153 | positive regulation of humoral immune response |
| GO:0016272 | 2,33E-04 | 0,0153 | prefoldin complex |
| GO:0055114 | 2,35E-04 | 0,0153 | oxidation-reduction process |
| GO:0030120 | 2,39E-04 | 0,0156 | vesicle coat |
| GO:0048518 | 2,91E-04 | 0,0188 | positive regulation of biological process |
| GO:0001974 | 3,16E-04 | 0,0204 | blood vessel remodeling |
| GO:0048268 | 3,24E-04 | 0,0208 | clathrin coat assembly |
| GO:0006417 | 3,33E-04 | 0,0213 | regulation of translation |
| GO:0035257 | 3,39E-04 | 0,0215 | nuclear hormone receptor binding |
| GO:0009150 | 3,52E-04 | 0,0223 | purine ribonucleotide metabolic process |
| GO:0051351 | 3,75E-04 | 0,0236 | positive regulation of ligase activity |
| GO:0002717 | 3,79E-04 | 0,0238 | positive regulation of natural killer cell mediated immunity |
| GO:0009144 | 3,87E-04 | 0,0242 | purine nucleoside triphosphate metabolic process |
| GO:0009141 | 3,96E-04 | 0,0247 | nucleoside triphosphate metabolic process |
| GO:0031575 | 4,14E-04 | 0,0256 | mitotic cell cycle G1/S transition checkpoint |
| GO:0002714 | 4,36E-04 | 0,0267 | positive regulation of B cell mediated immunity |
| GO:0002891 | 4,36E-04 | 0,0267 | positive regulation of immunoglobulin mediated immune response |
| GO:0009205 | 4,35E-04 | 0,0267 | purine ribonucleoside triphosphate metabolic process |
| GO:0000288 | 4,41E-04 | 0,0267 | nuclear-transcribed mRNA catabolic process, deadenylation-dependent decay |
| GO:0006521 | 4,41E-04 | 0,0267 | regulation of cellular amino acid metabolic process |
| GO:0043087 | 4,44E-04 | 0,0267 | regulation of GTPase activity |
| GO:0051247 | 4,42E-04 | 0,0267 | positive regulation of protein metabolic process |
| GO:2000112 | 4,60E-04 | 0,0276 | regulation of cellular macromolecule biosynthetic process |
| GO:0031324 | 4,73E-04 | 0,0283 | negative regulation of cellular metabolic process |
| GO:0043901 | 5,01E-04 | 0,0296 | negative regulation of multi-organism process |
| GO:0048147 | 5,01E-04 | 0,0296 | negative regulation of fibroblast proliferation |
| GO:0051437 | 5,01E-04 | 0,0296 | positive regulation of ubiquitin-protein ligase activity involved in mitotic cell cycle |
| GO:0031145 | 5,04E-04 | 0,0296 | anaphase-promoting complex-dependent proteasomal ubiquitin-dependent protein catabolic process |
| GO:0033124 | 5,11E-04 | 0,0299 | regulation of GTP catabolic process |
| GO:0009259 | 5,20E-04 | 0,0303 | ribonucleotide metabolic process |
| GO:0051439 | 5,57E-04 | 0,0323 | regulation of ubiquitin-protein ligase activity involved in mitotic cell cycle |
| GO:0009199 | 6,11E-04 | 0,0353 | ribonucleoside triphosphate metabolic process |
| GO:0002861 | 6,22E-04 | 0,0354 | regulation of inflammatory response to antigenic stimulus |
| GO:0043903 | 6,22E-04 | 0,0354 | regulation of symbiosis, encompassing mutualism through parasitism |
| GO:0046934 | 6,22E-04 | 0,0354 | phosphatidylinositol-4,5-bisphosphate 3-kinase activity |
| GO:0052813 | 6,22E-04 | 0,0354 | phosphatidylinositol bisphosphate kinase activity |
| GO:0006796 | 6,37E-04 | 0,0361 | phosphate-containing compound metabolic process |
| GO:0006367 | 6,50E-04 | 0,0367 | transcription initiation from RNA polymerase II promoter |
| GO:0048193 | 6,55E-04 | 0,0368 | Golgi vesicle transport |
| GO:0006464 | 6,63E-04 | 0,0370 | cellular protein modification process |
| GO:0006793 | 6,69E-04 | 0,0370 | phosphorus metabolic process |
| GO:0015992 | 6,67E-04 | 0,0370 | proton transport |
| GO:0036211 | 6,63E-04 | 0,0370 | protein modification process |
| GO:0043412 | 6,70E-04 | 0,0370 | macromolecule modification |
| GO:0000209 | 6,77E-04 | 0,0371 | protein polyubiquitination |
| GO:0051436 | 6,76E-04 | 0,0371 | negative regulation of ubiquitin-protein ligase activity involved in mitotic cell cycle |
| GO:0019899 | 7,00E-04 | 0,0382 | enzyme binding |
| GO:0006754 | 7,11E-04 | 0,0387 | ATP biosynthetic process |
| GO:0006511 | 7,18E-04 | 0,0389 | ubiquitin-dependent protein catabolic process |
| GO:0006357 | 7,32E-04 | 0,0395 | regulation of transcription from RNA polymerase II promoter |
| GO:0071779 | 7,36E-04 | 0,0396 | G1/S transition checkpoint |
| GO:0051246 | 7,56E-04 | 0,0406 | regulation of protein metabolic process |
| GO:0019941 | 7,63E-04 | 0,0407 | modification-dependent protein catabolic process |
| GO:0048199 | 7,68E-04 | 0,0409 | vesicle targeting, to, from or within Golgi |
| GO:0004386 | 7,84E-04 | 0,0414 | helicase activity |
| GO:0048002 | 7,84E-04 | 0,0414 | antigen processing and presentation of peptide antigen |
| GO:0033363 | 8,41E-04 | 0,0441 | secretory granule organization |
| GO:0036092 | 8,41E-04 | 0,0441 | phosphatidylinositol-3-phosphate biosynthetic process |
| GO:0006352 | 8,52E-04 | 0,0445 | DNA-dependent transcription, initiation |
| GO:0000291 | 8,70E-04 | 0,0450 | nuclear-transcribed mRNA catabolic process, exonucleolytic |
| GO:0007062 | 8,70E-04 | 0,0450 | sister chromatid cohesion |
| GO:0043928 | 8,70E-04 | 0,0450 | exonucleolytic nuclear-transcribed mRNA catabolic process involved in deadenylation-dependent decay |
| GO:0006163 | 8,94E-04 | 0,0461 | purine nucleotide metabolic process |
| GO:0003743 | 9,06E-04 | 0,0465 | translation initiation factor activity |
| GO:0005665 | 9,23E-04 | 0,0471 | DNA-directed RNA polymerase II, core complex |
| GO:0035035 | 9,23E-04 | 0,0471 | histone acetyltransferase binding |
| GO:0006446 | 9,40E-04 | 0,0476 | regulation of translational initiation |
| GO:0016469 | 9,40E-04 | 0,0476 | proton-transporting two-sector ATPase complex |
| GO:0032270 | 9,42E-04 | 0,0476 | positive regulation of cellular protein metabolic process |
| GO:0043632 | 9,89E-04 | 0,0498 | modification-dependent macromolecule catabolic process |
| GO:0010628 | 9,95E-04 | 0,0498 | positive regulation of gene expression |
| GO:0051438 | 9,98E-04 | 0,0498 | regulation of ubiquitin-protein ligase activity |
