# Supplementary material for: Different Blood Cell-Derived Transcriptome Signatures in Cows Exposed to Vaccination Pre- or Postpartum
Source: PLoS One. 2015 Aug 28;10(8):e0136927. doi: 10.1371/journal.pone.0136927 (PMC4552870; doi:10.1371/journal.pone.0136927)
Supplement: S5 Table — Significance threshold: q<0.05. (DOCX) [file pone.0136927.s005.docx]

Additional file 5, Table S5: GO term enrichment in differentially-expressed genes in response to vaccination after calving (FDR, q< 0.05)

| Category | Over represented (p-value) | q-value | GO Term |
| --- | --- | --- | --- |
| GO:0030099 | 9.84E-09 | 0.0001 | myeloid cell differentiation |
| GO:0030218 | 5.19E-08 | 0.0004 | erythrocyte differentiation |
| GO:0034101 | 9.52E-08 | 0.0005 | erythrocyte homeostasis |
| GO:0048513 | 1.32E-07 | 0.0005 | organ development |
| GO:0015669 | 8.44E-07 | 0.0021 | gas transport |
| GO:0030097 | 7.03E-07 | 0.0021 | hemopoiesis |
| GO:0048872 | 1.58E-06 | 0.0033 | homeostasis of number of cells |
| GO:0048534 | 1.90E-06 | 0.0035 | hemopoietic or lymphoid organ development |
| GO:0031674 | 3.46E-06 | 0.0056 | I band |
| GO:0002520 | 3.91E-06 | 0.0057 | immune system development |
| GO:0005576 | 4.93E-06 | 0.0063 | extracellular region |
| GO:0007275 | 5.15E-06 | 0.0063 | multicellular organismal development |
| GO:0002376 | 6.78E-06 | 0.0077 | immune system process |
| GO:0048731 | 7.51E-06 | 0.0079 | system development |
| GO:0070301 | 1.03E-05 | 0.0100 | cellular response to hydrogen peroxide |
| GO:0005344 | 1.18E-05 | 0.0109 | oxygen transporter activity |
| GO:0005833 | 2.36E-05 | 0.0204 | hemoglobin complex |
| GO:0030018 | 2.61E-05 | 0.0213 | Z disc |
| GO:0030017 | 3.12E-05 | 0.0241 | sarcomere |
| GO:0030154 | 3.69E-05 | 0.0268 | cell differentiation |
| GO:0032502 | 3.84E-05 | 0.0268 | developmental process |
| GO:0044421 | 4.63E-05 | 0.0308 | extracellular region part |
| GO:0004896 | 5.25E-05 | 0.0322 | cytokine receptor activity |
| GO:0009897 | 5.30E-05 | 0.0322 | external side of plasma membrane |
| GO:0044449 | 5.49E-05 | 0.0322 | contractile fiber part |
| GO:0048869 | 6.28E-05 | 0.0354 | cellular developmental process |
| GO:0048856 | 7.23E-05 | 0.0392 | anatomical structure development |
| GO:0030016 | 8.41E-05 | 0.0440 | myofibril |
| GO:0043034 | 9.10E-05 | 0.0460 | costamere |
| GO:0034614 | 9.87E-05 | 0.0469 | cellular response to reactive oxygen species |
| GO:0005602 | 1.15E-04 | 0.0469 | complement component C1 complex |
| GO:0005914 | 1.12E-04 | 0.0469 | spot adherens junction |
| GO:0015670 | 1.12E-04 | 0.0469 | carbon dioxide transport |
| GO:0030185 | 1.12E-04 | 0.0469 | nitric oxide transport |
| GO:0031720 | 1.14E-04 | 0.0469 | haptoglobin binding |
| GO:0043292 | 1.15E-04 | 0.0469 | contractile fiber |
