# Supplementary material for: Different Blood Cell-Derived Transcriptome Signatures in Cows Exposed to Vaccination Pre- or Postpartum
Source: PLoS One. 2015 Aug 28;10(8):e0136927. doi: 10.1371/journal.pone.0136927 (PMC4552870; doi:10.1371/journal.pone.0136927)
Supplement: S8 Table — Significance threshold: q<0.05. (DOCX) [file pone.0136927.s008.docx]

**Additional file 8 - Table S8. Significantly affected KEGG pathways of differentially expressed genes in response to vaccination prior to calving** (p< 0.05)

| KEGG category | Overrepresented (p-value) | KEGG pathway |
| --- | --- | --- |
| 3010 | 1.38E-40 | Ribosome |
| 5016 | 1.63E-22 | Huntington's disease |
| 5012 | 2.90E-21 | Parkinson's disease |
| 190 | 1.45E-19 | Oxidative phosphorylation |
| 5010 | 6.70E-19 | Alzheimer's disease |
| 4260 | 0.0001 | Cardiac muscle contraction |
| 4150 | 0.0002 | mTOR signalling pathway |
| 3050 | 0.0004 | Proteasome |
| 5215 | 0.0009 | Prostate cancer |
| 5213 | 0.0009 | Endometrial cancer |
| 4120 | 0.0014 | Ubiquitin mediated proteolysis |
| 5323 | 0.0015 | Rheumatoid arthritis |
| 5211 | 0.0025 | Renal cell carcinoma |
| 4940 | 0.0026 | Type I diabetes mellitus |
| 4510 | 0.0027 | Focal adhesion |
| 3018 | 0.0042 | RNA degradation |
| 4660 | 0.0065 | T cell receptor signalling pathway |
| 4810 | 0.0078 | Regulation of actin cytoskeleton |
| 5100 | 0.0110 | Bacterial invasion of epithelial cells |
| 5214 | 0.0167 | Glioma |
| 5221 | 0.0176 | Acute myeloid leukemia |
| 4012 | 0.0216 | ErbB signalling pathway |
| 4662 | 0.0216 | B cell receptor signalling pathway |
| 4650 | 0.0240 | Natural killer cell mediated cytotoxicity |
| 5218 | 0.0269 | Melanoma |
| 3013 | 0.0275 | RNA transport |
| 4062 | 0.0302 | Chemokine signalling pathway |
| 4973 | 0.0336 | Carbohydrate digestion and absorption |
| 3060 | 0.0370 | Protein export |
| 4060 | 0.0371 | Cytokine-cytokine receptor interaction |
| 5223 | 0.0376 | Non-small cell lung cancer |
| 4370 | 0.0376 | VEGF signalling pathway |
| 5310 | 0.0399 | Asthma |
| 3008 | 0.0417 | Ribosome biogenesis in eukaryotes |
| 4960 | 0.0471 | Aldosterone-regulated sodium reabsorption |
