# Supplementary material for: Different Blood Cell-Derived Transcriptome Signatures in Cows Exposed to Vaccination Pre- or Postpartum
Source: PLoS One. 2015 Aug 28;10(8):e0136927. doi: 10.1371/journal.pone.0136927 (PMC4552870; doi:10.1371/journal.pone.0136927)
Supplement: S9 Table — Significance threshold: q<0.05. (DOCX) [file pone.0136927.s009.docx]

**Additional file 9 - Table S9. Significantly affected KEGG pathways of differentially expressed genes in response to vaccination after to calving** (p< 0.05)

| KEGG category | Overrepresented (p-value) | KEGG pathway |
| --- | --- | --- |
| 4610 | 0.0024 | Complement and coagulation cascades |
| 5020 | 0.0038 | Oxidative phosphorylation |
| 4640 | 0.0039 | Hematopoietic cell lineage |
| 5144 | 0.0055 | Malaria |
| 4964 | 0.0094 | Proximal tubule bicarbonate reclamation |
| 4080 | 0.0147 | Neuroactive ligand-receptor interaction |
| 4630 | 0.0237 | Jak-STAT signalling |
| 4060 | 0.0273 | Cytokine-cytokine receptor interaction |
| 5143 | 0.0308 | African trypanosomiasis |
| 4966 | 0.0354 | Collecting duct acid secretion |
| 5150 | 0.0456 | Staphylococcus aureus infection |
